# Revitalization of Total Petroleum Hydrocarbon Contaminated Soil Remediated by Landfarming

**DOI:** 10.3390/toxics10030147

**Published:** 2022-03-19

**Authors:** Woo-Chun Lee, Jong-Hwan Lee, Sang-Hun Lee, Sang-Woo Lee, Ji-Hoon Jeon, Sang-Hwan Lee, Soon-Oh Kim

**Affiliations:** 1 Department of Geology and Research Institute of Natural Science (RINS), Gyeongsang National University (GNU), Jinju 52828, Korea; riveras@gnu.ac.kr (W.-C.L.); 4sssis@daum.net (J.-H.L.); ss199573@naver.com (S.-H.L.); lswenv@hanmail.net (S.-W.L.); jeonjh@gnu.ac.kr (J.-H.J.); 2 Technical Research Institute, Mine Reclamation Corporation, Wonju 26464, Korea; soillsf@gmail.com

**Keywords:** soil health, revitalization, total petroleum hydrocarbon, landfarming

## Abstract

Soil health deteriorates through the contamination and remediation processes, resulting in the limitation of the reuse and recycling of the remediated soils. Therefore, soil health should be recovered for the intended purposes of reuse and recycling. This study aimed to evaluate the applicability and effectiveness of several amendments to revitalize total petroleum hydrocarbon contaminated soils remediated by the landfarming process. Ten inorganic, organic, and biological amendments were investigated for their dosage and duration, and nine physicochemical, four fertility, and seven microbial (soil enzyme activity) factors were compared before and after the treatment of amendments. Finally, the extent of recovery was quantitatively estimated, and the significance of results was confirmed with statistical methods, such as simple regression and correlation analyses assisted by principal component analysis. The landfarming process is considered a somewhat environmentally friendly remediation technology to minimize the adverse effect on soil quality, but four soil properties—such as water holding capacity (WHC), exchangeable potassium (Ex. K), nitrate-nitrogen (NO_3_-N), available phosphorus (Av. P), and urease—were confirmed to deteriorate through the landfarming process. The WHC was better improved by organic agents, such as peat moss, biochar, and compost. Zeolite was evaluated as the most effective material for improving Ex. K content. The vermicompost showed the highest efficacy in recovering the NO_3_-N content of the remediated soil. Chlorella, vermicompost, and compost were investigated for their ability to enhance urease activity effectively. Although each additive showed different effectiveness according to different soil properties, their effect on overall soil properties should be considered for cost-effectiveness and practical implementation. Their overall effect was evaluated using statistical methods, and the results showed that compost, chlorella, and vermicompost were the most relevant amendments for rehabilitating the overall health of the remediated soil for the reuse and/or recycling of agricultural purposes. This study highlighted how to practically improve the health of remediated soils for the reuse and recycling of agricultural purposes.

## 1. Introduction

Humanity has considered soil an invaluable resource due to its intrinsic functions, such as crop production, groundwater recharge, and serving diverse organisms’ habitats. Furthermore, the other functions—such as carbon storage and maintaining biodiversity—have recently been emphasized [1,2]. However, indiscreet activities have increased soil contamination, and economical and practical technologies have been continuously developed to remediate the contaminated soils [3,4,5,6]. Even though the remediation processes can reduce contamination levels, soil health simultaneously deteriorates. As a result, reuse and recycling of remediated soil are constricted for relevant and intended land usage [7,8,9]. Herein, the difference between the definitions of soil health and quality needs to be mentioned. Soil quality focuses more on the soil’s capacity to meet defined human needs such as the growth of a particular crop, whilst soil health focuses more on the soil’s continued capacity to sustain plant growth and maintain its functions [10]. However, in a program to assess and monitor soil quality in Canada, the term soil quality was used interchangeably with soil health [11]. Therefore, the terms are considered equivalent hereafter.

Among diverse remediation technologies of contaminated soils, landfarming, soil washing, and thermal desorption (treatment) have been most widely used in Korea [12].

Landfarming has usually been applied to remediate soils contaminated with hydrocarbon compounds (fossil fuels) leaked from gas stations; oil storage facilities, including pipelines; and oil spill accidents [13]. It has been mostly implemented by ex-situ treatment in Korea [14,15]. Even though the contamination levels are lessened by the process, the soil health is degraded after treatment due to the decrease in fertility and the changes of pH, the disturbance of the native community of microorganisms, and the imbalance of soil enzymes. Accordingly, in addition to the contamination period, the remediation process deteriorates the health of the soil [16,17,18]. As a result, the reuse and recycling of the remediated soils are limited. The quantity of remediated soils has gradually increased in Korea: it was reported to have increased 100 times from 2005 to 2016 [19]. However, most remediated soils are stored in treatment plants for long periods because of the limited reuse/recycling. A small quantity has been used as fill material for the construction of roads and buildings. To recover the health of remediated soils, the change in their properties and functions through the treatment process should be scrutinized, and then relevant technologies need to be developed based on the previous results. Subsequently, it is necessary to confirm if the remediated soil is revitalized for the intended land use via diagnosing the various indices related to soil health. A variety of materials have been used to improve soil quality, and they can be categorized into three groups based on their origin—namely, inorganic, organic, and biological amendments [16]. Among them, organic additives have been most frequently used to enhance the activity of soil microorganisms [20,21]. Recently, numerous studies have focused on applying biological amendments to rehabilitate soils because of their direct effects on soil quality [22,23]. The direct medication of environmentally friendly and economical biological agents—such as earthworm casting (vermicompost), plants, and microorganisms—can promote favorable environments for diverse soil organisms and improve soil health [24,25,26,27,28]. Inorganic materials—such as fly ash, zeolite, and bentonite—are known to improve available nitrogen and phosphorus, and lime is effective in adjusting pH and immobilizing heavy metal contaminants [29,30,31,32,33,34].

There has been little research on the restoration of soils remediated by landfarming, but some studies were conducted to characterize the deterioration of soil quality and the ecological toxicity of residual contaminants after the landfarming process [35,36]. Landfarming does not affect physical properties of soil as much as the other remediation technologies because of the mechanism of microorganism decomposition, but it is known that it influences the properties related to the activity of microorganisms, such as pH, nutrients (soil organic matter, available phosphorus, nitrate-nitrogen, etc.), and soil enzymes [37]. Yi et al., (2012) reported that exchangeable K/Mg and Ca decreased and increased, respectively, after the application of the landfarming process [17]. In addition, it was reported that the changing tendency of soil enzymes was different depending on their type: protease and arylsulfatase were increased, but acid phosphatase decreased during the landfarming process [18,35]. Hamdi et al. (2007) showed that the residual toxicity of polyaromatic hydrocarbon (PAH) was not observed, and soil pH was decreased after landfarming [38]. Otherwise, most research focused on improving landfarming by adding different nutrients and additives, including microorganisms [39,40]. In short, landfarming, a biological process, seems to deteriorate soil health less than other physicochemical technologies, and there have been few studies on the restoration of soil health after remediation. However, it is necessary to elucidate the change of various properties of soils after landfarming and to develop an effective revitalization technology relevant for the intended purposes of reuse and recycling.

This study aimed to evaluate the applicability and effectiveness of several amendments to revitalize the total petroleum hydrocarbon contaminated soils remediated by the landfarming process. To this end, several physicochemical, fertility, and microbial (soil enzyme) factors were compared before and after the landfarming process to extract the deteriorated indices of soil health. Subsequently, the applicability of 10 inorganic, organic, and biological amendments was evaluated according to their type, dosage, and duration. Finally, the extent of recovery was quantitatively estimated, and the significance of results was confirmed with statistical methods, such as simple regression and correlation analyses through principal component analysis (PCA).

## 2. Materials and Methods

### 2.1. Soil, Remediation Technology, and Analytical Methods

The soil used in this study was contaminated with total petroleum hydrocarbon (TPH) due to leakage from vehicles and heating fuels within a closed military site located in Dongducheon City, Korea, and remediated by ex situ landfarming. The remediation process consisted of adjusting pH and supplying water, nutrients, and air to stimulate the activity of aerobic microbes. Three types of soil samples—non-contaminated (NC), contaminated (CS), and remediated (RS)—were taken to compare their properties and characterize the deterioration through the remediation process. All the soils belong to Cambisoils according to the WRB soil classification system. The soil samples were dried and pretreated with a #10 sieve with a mesh size of smaller than 2 mm before analyzing TPH concentrations, physicochemical factor including seven properties (water holding capacity (WHC), soil texture, aggregate stability (AS), pH, electrical conductivity (EC), concentrations of exchangeable cations (Ex. Na, K, Mg, and Ca), cation exchange capacity (CEC)), fertility factor represented by four properties (soil organic matter (SOM), total nitrogen (T-N), nitrate-nitrogen (NO_3_-N), available phosphorus (Av. P)), and microbial factor designated by the activity of seven soil enzymes (β-glucosidase (BG), N-acetyl-β-glucosaminidase (NAG), urease (Ure), acid phosphatase (ACP), alkaline phosphatase (ALP), arylsulfatase (ARS), and dehydrogenase (DHA)). The details of each analysis are given in the Supplementary Materials (Table S1) [41,42,43,44,45,46,47,48,49,50]. All the analyses were done in triplicate.

### 2.2. Experimental Design

Several sets of experiments were conducted to evaluate the applicability of three inorganic amendments (zeolite, gypsum, and dolomitic lime), three organic materials (peat moss, compost, and biochar), three biological agents (earthworm casting (vermicompost), chlorella, effective microorganism), and a composite fertilizer. All the materials were for commercial sale. The reasons for selecting those materials were that they have been widely used as soil improvement additives and are cheap and environmentally friendly, except for composite fertilizer. The details of each material are given in Table S2, and their primary properties determined by the methods given in Table S1 are presented in Table 1. The composite fertilizer used in farmlands is composed of N (11%), P (7%), K (10%), MgO (2%), and B (1.2%), and it was selected as the prime material to compare the efficacies of the other amendments.

The dosages of each agent were 1.0, 2.5, 5.0, 7.5, and 10% (*wt*/*wt*), and they were homogeneously mixed with 1.5 kg of the RS. Generally, soil improvement additives are used in farmlands at a level of less than 3%. However, the quality of the RS deteriorated much more than that of farmlands, and relatively higher dosages were tested in the experiments. Except for the effective microorganism agent, the materials were powder and easily blended with soils. The effective microorganism materials were obtained in tablet form, and they were dissolved in distilled water (4.5 g/L) before addition to soils. Soil moisture content was adjusted with the maximum WHC of 50%, and then the treated soils were aged for 10 weeks. All the treatments were done in triplicate. The samples of treated soils were taken three times (2, 6, and 10 weeks) after adding amendments to quantify the improvement of deteriorated properties. The RS without adding materials was used as a control, and water was only added to maintain the equivalent moisture content during the experimental period. Depending on the types of amendments, different properties were improved, and different properties were monitored for each material (Table 2). In the case of peat moss treatment, only seven physicochemical properties and SOM were monitored because it has been reported that it could not improve the other properties [51,52]. The improvement of RS aimed for two purposes of reuse and/or recycling—namely, agricultural and landscape (forest field) uses. In that sense, the tendency of deterioration through the remediation process was taken into consideration.

### 2.3. Statistical Analyses

#### 2.3.1. Regression Analyses

The significance of the results was confirmed using statistical methods, and the extent of improvement was estimated according to the type and dosage of additives and duration. A simple regression analysis was used to assess the linearity between the dosage of each material and the properties of treated soils and to investigate the significance of the slopes (Figure S1). Regression equations included in the R program package were used for the simple regression analysis, and significant differences *(p*-values) were distinguished in the three ranges, such as *p* < 0.05, < 0.01, and < 0.001. Based on the *p*-values and the slopes, the effectiveness of each amendment was compared.

#### 2.3.2. Correlation Analyses through PCA

For the most effective amendments, the interrelationship between the improved and other properties of soils was investigated with correlation coefficients using the SigmaStat 4.0 program of the Sigmaplot 14.0 package (Systat Software Inc., San Jose, CA, USA). The correlation between amendment type and effect was evaluated using PCA. The standard context for correlation analysis involves a correlation coefficient (r) designating the direction and intensity between two variables. However, there are some limitations of the analysis, such as the nonlinearity between variables not being explained, the averages and standard deviations of data not being reflected, and the correlation between variables not being perfectly interpreted due to the existence of uncorrected outliers. It is difficult that most datasets taken from natural and man-made environments show normal (Gaussian) distribution, but PCA as a descriptive tool needs no distributional assumptions and, as such, is very much an adaptive exploratory method which can be used on numerical data of various types [53]. So far, PCs have been presented as linear combinations of the (centered) original variables. However, the properties of PCA have some undesirable features when these variables have different units of measurement. Three durations (2, 6, and 10 weeks) and five dosages (1.0, 2.5, 5.0, 7.5, and 10 wt %) for the total of 20 soil properties monitored had different units, and the directionalities and magnitudes of relationship, reliance, and similarity between independent variables (durations and dosages) and dependent variables (soil properties) are different [54]. For these reasons, instead of conventional correlation analyses, accordingly, PCA was conducted to improve reliability and significance level. The PCA was carried out using the SigmaStat 4.0 program, and this approach was widely used in the previous studies [53]. To overcome the fact that PCA is defined by a criterion (variance) that depends on units of measurement implies that PCs based on the covariance matrix *S* will change if the units of measurement on one or more of the variables change, it is common practice to begin by standardizing the variables. Each data value *x_ij_* is both centered and divided by the standard deviation *s_j_* of the *n* observations of variable *j* [53].

(1) $$z_{ij}=\frac{x_{ij}-\bar{x_{j}}}{s_{j}}$$

Thus, the initial data matrix X is replaced with the standardized data matrix Z, whose *j*-th column is vector *z_j_* with the *n* standardized observations of variable *j*.

PCA was conducted for the three most effective additives selected in Section 3.3—namely, compost, chlorella, and vermicompost—and PCs, their proportion, and cumulative proportion were estimated. The normalization of the correlation matrix was undertaken following the method explained by Jolliffe and Cadima (2016) [53]. Correlation matrix PCs are invariant to linear changes in units of measurement and are therefore the appropriate choice for datasets where different changes of scale are conceivable for each variable. Some statistical software assumes by default that a PCA means a correlation matrix PCA and, in some cases, the normalization used for the vectors of loadings *a_k_* of correlation matrix PCs is not the standard *a′_k_a_k_* = 1. In a correlation matrix PCA, the coefficient of correlation between the *j*-th variable and the *k*th PC is given by

(2) $$r_{var_{j},PC_{k}}=\sqrt{\lambda_{k}a_{jk}}$$

Thus, if the normalization *ã′_k_ã_k_* = *λ_k_* is used instead of *a′_k_a_k_* = 1, the coefficients of the new loading vectors *ã_k_* are the correlations between each original variable and the *k*th PC. Refer to Jolliffe and Cadima (2016) for a more detailed explanation [53].

## 3. Results and Discussion

### 3.1. Deterioration of Soil Quality through the Landfarming Process

The TPH concentration of NS showed a lower level than the detection limit (50 mg/kg) and those of CS and RS were 6140 ± 332 and 172 ± 10 mg/kg, respectively, indicating that the landfarming process effectively removed the contaminants. The analytical results of properties for each soil are given in Table 3, and they were obtained before treatment (addition of amendments). To judge which properties were degraded, two criteria for agricultural and landscape (forest field) uses were referred to, as suggested by the National Institute of Agricultural Sciences (NAAS) of Korea and the Korean Institute of Landscape Architecture (KILA), respectively [55,56]. The criteria are presented in Tables S3 and S4. In the case of properties without any criteria, judgement was based on those of the NS.

#### 3.1.1. Physicochemical Factor

Some physicochemical properties deteriorated through the remediation process. The water holding capacity (WHC) is the soil moisture content available to plants and directly influences crop productivity [57]. The WHC of the RS was measured at 24.8% and decreased slightly, compared with those of the NS and CS (Table 3). The soil texture was determined using the method suggested by the USDA, and the NS and CS/RS showed somewhat different textures—such as loamy sand and sandy loam, respectively—due to their heterogeneities (Table 3 and Figure S2). The AS is a representative physical property of soil and significantly affects plant growth. It is well known that the decrease in the AS results in a structural change from aggregates (peds) to single grains and brings about detrimental effects on root growth and the absorption of water and nutrients [58]. The AS (44.2%) of the RS was increased by 34% through the remediation process, showing a positive effect of landfarming (Table 3). The soil pH is a crucial factor in controlling the transport of substances and the activity of microorganisms in soil environments [59]. The pH of RS was 6.0 and decreased by 1.0, compared with the CS, and met the agricultural criterion (5.5–7.0) and the landscape standard (4.5–8.0). The electrical conductivity (EC) measuring the salinity of soils is a critical factor because it is vulnerable to change through remediation processes. The EC of the RS appeared to be 70.2 μS/cm, and the landfarming process did not seem to affect it. It also met two criteria for reuse and recycling in agricultural and landscape uses (≤2000 μS/cm) (Table 3). Exchangeable cations (Ex. Na, K, Mg, and Ca) in soils supply essential nutrients for crop production, and their relevant contents are suggested for agricultural and landscape uses, except for Ex. Na. Several kinds of nutrients were supplied to activate microbes during the landfarming process, and oversupplying causes an increase in the soil salinity. The contents of Ex. Na, Mg, and Ca did not seem to be problematic, but those of Ex. K appeared to be lower than the criterion for reuse and recycling for landscape (forest field) (Table 3). Moreover, the concentrations of Ex. Mg and Ca were decreased through the remediation process. Quilchano and Marañón (2002) reported that the decrease in Ex. Mg and Ca might reduce dehydrogenase activity, and it was likely that their lower contents adversely affected soil enzyme activities [60]. A higher cation exchange capacity (CEC) means a more considerable quantity of cationic nutrients available to plants, and the fertility of soils is increased with the increase in CEC [31,61]. Sometimes in an actual situation, the fertility is not markedly increased with the increase in CEC, but a higher CEC potentially enhances the fertility. The CEC of RS was 11.6 cmolc/kg, and there was no remarkable difference between before and after remediation. The CEC of the RS was determined to satisfy two criteria for reuse and recycling (Table 3). In short, the WHC deteriorated through the landfarming process, and it needed to be improved for the reuse and recycling. In addition, the Ex. K was not degraded during the remediation process, but that of the RS did not meet the criterion for landscape (forest field) use. Hence, it should be improved for the reuse and recycling of landscape purposes.

#### 3.1.2. Fertility Factor

Fertility factors are most crucial for the crop productivity of soils, and the recommended contents of soil organic matter (SOM) and available phosphorus (Av. P) are included in the two criteria for agricultural use and forest field. The SOM enhances the formation of aggregate structure, and thereby increases air permeability of soils. In addition, the SOM functions not only to buffer an abrupt change of soil pH and EC, but also serves as feed for soil microorganisms [62]. The SOM content of the RS was measured at 3.5% and was higher than those of the NS and CS due to nutrient supply and microbe activities during the landfarming process. It met the two criteria (Table 3). On the other hand, the total nitrogen (T-N) content of the RS appeared to be 638 mg/kg, similar to the level of the NS due to nutrient supply (Table 3). In the case of the CS, the T-N was 149 mg/kg, which was much lower than the recommendation for landscape use, but it was improved to meet the standard during the remediation process. The nitrate nitrogen (NO_3_-N) content of the RS was determined to be 1.74 mg/kg. It deteriorated during the remediation (Table 3) and should be enhanced for reuse and recycling. It is speculated that the NO_3_-N was decreased as a result of the consumption of microbes and the denitrification by soil enzymes, such as urease [63], although the T-N content increased to the level of the NS due to the nutrient supply. The Av. P content was measured at 16.02 mg/kg in the RS. Even though it was considerably increased, compared with the NS and CS, it did not still meet the two criteria and should be improved. Among the fertility factors, in summary, the contents of NO_3_-N and Av. P needed to be enhanced for reuse and recycling.

#### 3.1.3. Microbial Factor

The overall performance and duration of the landfarming process are dependent on microorganisms’ activity, and participant soil enzymes play a particularly crucial role. For this reason, seven soil enzymes mediating the cycling of primary nutrients—such as C, N, P, S, and H—were monitored. Except for urease, most of them were activated by the landfarming process, and their concentrations were compared between the NS, CS, and RS (Table 3). The activities of β-glucosidase (BG) and arylsulfatase (ARS) were the most activated enzymes. Yi et al. (2016) reported a remarkable increase in the activities of those two enzymes during landfarming [18]. In the case of urease (Ure) and alkaline phosphatase (ALP), their concentrations were measured as lower in the CS than in the NS, which might be caused by the detrimental effects of hydrocarbon compounds [64]. Furthermore, the Ure content was remarkably decreased by contamination and was not recovered through the remediation process, indicating that it should be improved for reuse and recycling.

### 3.2. Evaluation of the Effectiveness of Individual Additive

#### 3.2.1. Effects on the Physicochemical Factor

**Figure 1:**
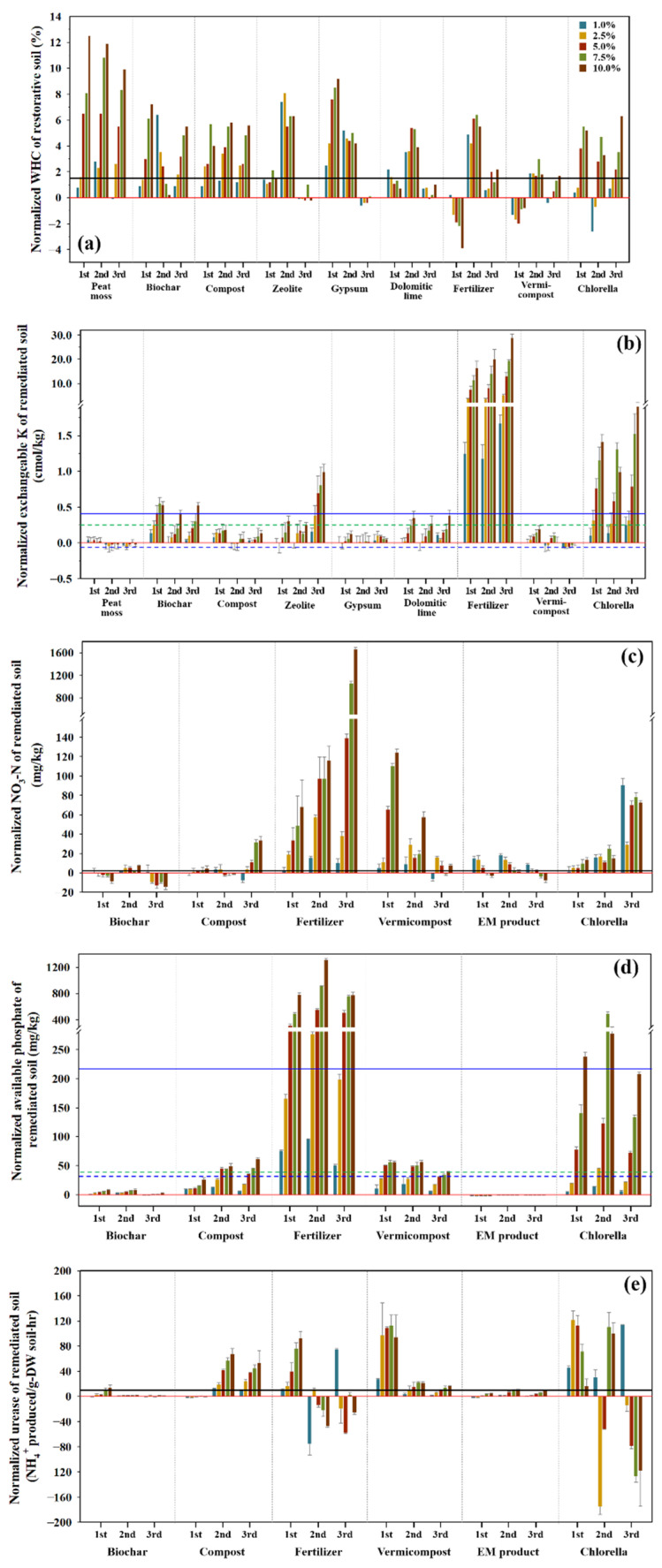
Changes in soil properties according to the dosage of each amendment and the duration of treatment. (**a**) Water holding capacity (WHC), (**b**) exchangeable K (Ex. K), (**c**) nitrate-nitrogen (NO_3_-N), (**d**) available phosphorus (Av. P), and (**e**) Urease (Ure). The first, second, and third periods mean after 2, 6, 10 weeks of treatment. The blue and blue dotted lines are the upper and lower limits of the criteria of soil quality standard for agricultural uses, respectively. The green dotted line is the lower limit of the criteria of soil quality standard for landscape (forest field) uses and the black line is the properties of non-contaminated soil (NS). Each result was normalized by subtracting the values of remediated soil (RS) from the values of restored soil.

In total, 10 kinds of amendments were assessed for the revitalization of the landfarming remediated soil. Even though only five properties were degraded through the remediation process, many materials were evaluated because they exhibit different effects, and deteriorated properties could be improved by their combined efficacies. After the injection of each additive, the change of physicochemical, fertility, and microbial (soil enzyme) factors was monitored to evaluate the overall effects of amendments on the soil quality. The results were normalized by subtracting the measured values of control tests without injecting any amendments from those of treated soils (Figure 1, Figures S3, S5 and S6). First, peat moss was the most effective additive when looking into the effects of amendments on WHC. The WHC was increased with the increase in dosage of peat moss but seemed to slightly decrease with the increase in duration (Figure 1a). For all materials, a smaller dosage of about 2.5% seemed to be sufficient in improving the WHC to meet the level of the NS. Overall, the WHC was better improved by organic agents (peat moss, biochar, and compost) than the others. Inorganic materials—such as zeolite, gypsum, and dolomitic lime—enhanced the WHC until the sixth week (the second period) after adding them, but their effects abruptly decreased at the 10th week (the third period). This was caused by the decrease in their stability or dissolution as a result of the interaction with soil moisture as time went on. During the measurement of pH, EC, and AS, it was confirmed that gypsum and dolomitic lime were easily dissolved in the distilled water.

Soil texture was not changed during the remediation process, and it was not necessary to evaluate the effects of the amendments on the soil texture. However, the impact of additives on the change of soil texture was assessed. Overall, the treatment of amendments increased the proportion of sand content, and the higher the dosage was, the more definite the tendency observed (Figure S4). This might be caused by the sizes of amendments added. Most of the inorganic additives consisted of the sand size (Table 1), which was likely to affect the change of soil texture. Meanwhile, the sand content was gradually decreased after the sixth week (the second period), and the tendency was remarkable in the case of gypsum treatment (Figure S4f). This seemed to be due to the dissolution as mentioned above.

Compost was assessed to be the most effective in improving AS (Figure S3a). However, the other materials decreased the AS or showed irregular patterns according to the treatment duration. While the peat moss enhanced the WHC, it also decreased the AS. This might be attributed to the destruction of peds due to the peat moss’ effect of lowering soil pH [62,65].

In terms of the effects of amendments on soil pH, most of them increased it, except for peat moss, zeolite, and gypsum (Figure S3b). The effects of each material seemed to originate from their native pH (Table 1). In particular, the pH of chlorella (6.7) was only 0.7 higher than the RS (6.0), and it increased the soil pH in the order of larger than 1.0. This was caused by the fact that chlorella had a relatively higher content of exchangeable basic cations and actively affected soil enzymes (Table 1 and Figure S6) [66,67]. Even though biochar and dolomitic lime showed much higher pHs (>10), compost, dolomitic lime, and chlorella were remarkably effective in increasing soil pH. Conversely, peat moss (pH of 3.9) and gypsum (pH of 5.7) seemed to be effective in improving alkaline soils.

An abnormal higher EC adversely affects soil quality, and amendments should not be overused. Except for gypsum and composite fertilizer, dosages up to 10% seemed not to exceed the EC criteria (lower than 2000 µS/cm) (Figure S3c). The EC values of gypsum and compound fertilizer were measured at 8550 and 110,400 µS/cm, respectively (Table 1), and their treatment caused the criteria to be exceeded. As mentioned previously, the composite fertilizer was tested as a basic additive to compare relative efficacies of the other amendments, and its practical use is likely to be designed relevant to the type of crop rather than used as an improvement agent. In the case of gypsum, the soil EC was gradually decreased after the sixth week up to the level of criteria. The EC values of compost, chlorella, and earthworm casting (vermicompost) were 4470, 3510, and 3300 µS/cm, respectively (Table 1), but the soil EC was least increased in the compost treatment (Figure S3c), which was contributed to the adsorption and/or complexation of ions with organic matters included in the compost [62]. Despite a lower EC of chlorella, its treatment considerably increased soil EC (Figure S3c), which might be caused by the dissolution of ions due to the reactions with soil enzymes activated by chlorella [67].

Zeolite was evaluated to be the most effective material for improving the content of Ex. Na, except for fertilizer. Notably, the 10-week treatment considerably increased the Ex. Na (Figure S3d). Compost seemed to be next most effective in improving Ex. Na and its effect was continuous throughout the duration. The Ex. K content of the RS did not meet the criteria and needed to be enhanced for reuse and recycling (Table 3). The materials’ effectiveness was evaluated in the order of chlorella > zeolite > biochar (Figure 1b). The effect of chlorella was likely due to its higher content of Ex. K (Table 1). Chlorella, zeolite, and biochar were effective in improving Ex. K to meet two criteria, and in particular, the optimum dosage of chlorella was investigated to be 5.0%. The zeolite’s effect was manifested only after the 10th week, similar to the Ex. Na, which is likely due to zeolite being a crystalline substance and cations needing enough time to dissolve. The effects of a 10% dosage seemed to correspond to those of 1% fertilizer, indicating that their dosages were not excessive. As expected, the Ex. Mg content was most efficiently improved by dolomitic lime due to its higher Mg content, and its recovery power was comparable to that of composite fertilizer (Figure S3e). In addition, chlorella, vermicompost, compost, and zeolite seemed to be effective for Ex. Mg. Even though the effect was insignificant with a lower content of chlorella, the extent of improvement was considerably increased with the increase in its content. The effect of compost also was similar to chlorella. However, zeolite and vermicompost showed an opposite tendency: the effect of zeolite increased with the increase in duration, but vermicompost decreased according to the duration of treatment. Zeolite is a hydrous aluminosilicate and shows a behavior analogous to clay minerals. The cations adsorbed in its interfacial spaces between unit lattices and on the edges of structure can be exchanged with hydrogen and ammonium ions produced by microbial activity and soil enzyme activation [68]. This process is enhanced with time. As a result, the Ex. Mg content gradually increased with the increase in time of the zeolite treatment. On the contrary, vermicompost, primarily composed of earthworm excreta, stimulated microbe activity and soil enzymes immediately after addition to soils, and therefore its effect was mediated quickly, indicating that it can shorten the recovery time. The Ex. Ca content was significantly increased by adding gypsum, compost, dolomitic lime, and vermicompost (Figure S3f). The effect of gypsum (CaSO_4_·2H_2_O) on the improvement of Ex. Ca was demonstrated in the earlier duration due to its greater solubility. A dose of only 1% gypsum could enhance Ex. Ca to meet its criteria for agricultural and landscape uses. Dolomitic lime and compost contained a relatively higher Ca content, and they effectively enhanced the Ex. Ca content.

The effective agents for increasing the soil CEC were peat moss, compost, zeolite, and vermicompost. The efficacy of compost became considerable after the 10th week (the third period) (Figure S3g). The vermicompost tended to increase soil EC after the second week, remarkably decreased it at the sixth week, and again increased it after the 10th week, which was likely due to a higher content of Ex. Mg and Ca (Table 1) and alternate adsorption and desorption (Figure S3e,f). Zeolite showed a favorable effect on improving soil CEC due to its higher CEC, and peat moss also exhibited effective enhancement because it contains many functional groups, such as carboxyl, serving as adsorption sites of cations.

#### 3.2.2. Effects on the Fertility Factor

The SOM content, a crucial factor for crop growth, was most effectively enhanced by chlorella, compost, vermicompost, and peat moss, and the other materials also increased it (Figure S5a). Those four amendments contained the organic matter contents of 72.2%, 51.8%, and 47.1%, and 44.1%, respectively (Table 1), and chlorella and vermicompost exhibited effects corresponding with their dosages. However, peat moss and compost showed much higher effectiveness than their added levels, which seemed to be caused by the transformation of the original SOM to soil SOM due to the microbe activity and soil enzymes [69].

Peat moss, zeolite, gypsum, and dolomitic lime were excluded from assessment because they were expected not to improve the T-N content, and the other six materials were evaluated. Like the SOM, most of the amendments tested increased the T-N content to a different extent (Figure S5b). The T-N content was increased in the sequence of chlorella > vermicompost > compost > biochar, which was identical to that of their T-N contents (Table 1). EM agent showed little contribution to the improvement of T-N. NO_3_-N gradually deteriorated through contamination and remediation (Table 3), and it needed to be enhanced for reuse and recycling. Like the T-N amendments, six materials were tested to verify their effects on its improvement because the other amendments did not contain NO_3_-N. Particularly, chlorella and vermicompost were investigated to be effective in improving NO_3_-N with a much smaller dosage (1.5%). Fertilizer overwhelmingly enhanced the NO_3_-N content. Even though the NO_3_-N content of chlorella was much higher than that of vermicompost (Table 1), the latter showed higher efficacy in recovering the NO_3_-N content of soil (Figure 1c), resulting from the complexity of transformation of T-N and NH_4_-N to NO_3_-N.

The content of Av. P was considerably increased through the remediation process, but it still was lower than the recommended levels for reuse and recycling (Table 3). Accordingly, the effect of six materials on the enhancement of Av. P was assessed. Chlorella was the most effective amendment, but its improving power was not comparable to fertilizer at all. However, its dosage of 10% exceeded the upper limit of criteria, and caution was needed to avoid overuse (Figure 1d). Compost and vermicompost should be treated for longer than six weeks with a dosage of ≥6%. The other agents showed feeble or no effects. With the dosage of 5%, chlorella could improve Av. P to meet the criterion for agricultural use.

#### 3.2.3. Effects on the Microbial (Soil Enzyme) Factor

Soil enzymes control the cycling of primary nutrients, thereby supporting crop growth and playing a crucial role in soundly sustaining the soil ecosystem [70,71]. Their activities reflect soil fertility, and they have been used as crucial factors to evaluate soil quality [72]. The effects of six amendments on seven soil enzymes (BG, NAG, Ure, ACP, ALP, ARS, and DHA) were evaluated. Above all, BG acts as an enzyme to hydrolyze cellulose to glucose, and it functions by supplying nutrients essential for plant growth. Chlorella and compost were evaluated as the most effective amendments activating most soil enzymes, including BG (Figure S6a). Compost seemed to be excellent in promoting soil enzyme activities as well. NAG is one of three enzymes that decomposes chitin, which is more resistant than cellulose and significantly influences the cycle of C and N in soil environments [73]. Like BG, NAG was considerably activated by chlorella and compost, and vermicompost also showed a similar effect (Figure S6b). Ure is a soil enzyme participating in the N cycle and transforms organic substances containing N into inorganic N compounds, such as NH_4_^+^ [74]. The experimental results demonstrated that chlorella, vermicompost, and compost affected Ure activity, and their manner for Ure activation seemed to be very complicated, maybe due to the combined processes of nitrification and denitrification. Their small dosage of about 1.5% seemed to be effective in enhancing Ure. It is well known that ACP and ALP show opposite activities depending on pH conditions: the former and latter are activated in acid and alkaline environments, respectively [75,76]. The soil pH was 6.0 after remediation, as shown in Table 3, and was 5.0–7.0 (approximately neutral pH) after treating with amendments (Figure S3b). Therefore, the activities of both enzymes exhibited a similar tendency (Figure S6c,d). Chlorella, compost, and vermicompost effectively improve their activities. Sulfur of 90–98% is known to exist in organic forms in soil environments, and sulfate ester occupies 30–75% organic S. ARS hydrolyzes this organic S and transforms it into inorganic forms [77,78]. Like the other enzymes, ARS was effectively activated by chlorella, vermicompost, and compost (Figure S6e). However, the vermicompost’s effect markedly diminished over time. DHA is an indicator of the oxidative metabolism and microbiological activity in soil environments, and its activation is directly related to microorganism activity [79,80]. The effect of each amendment on the DHA activation was observed to be highly different depending on the type. Chlorella and compost showed the best effect, and vermicompost was somewhat effective (Figure S6f).

### 3.3. Regression Analyses

**Figure 2:**
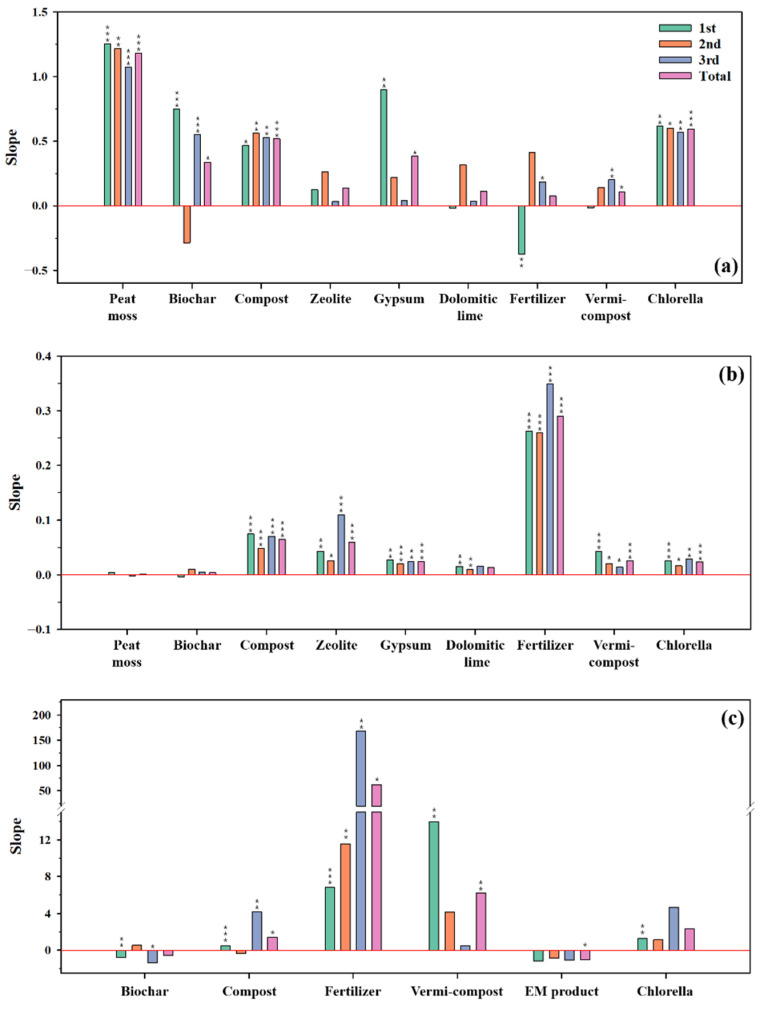

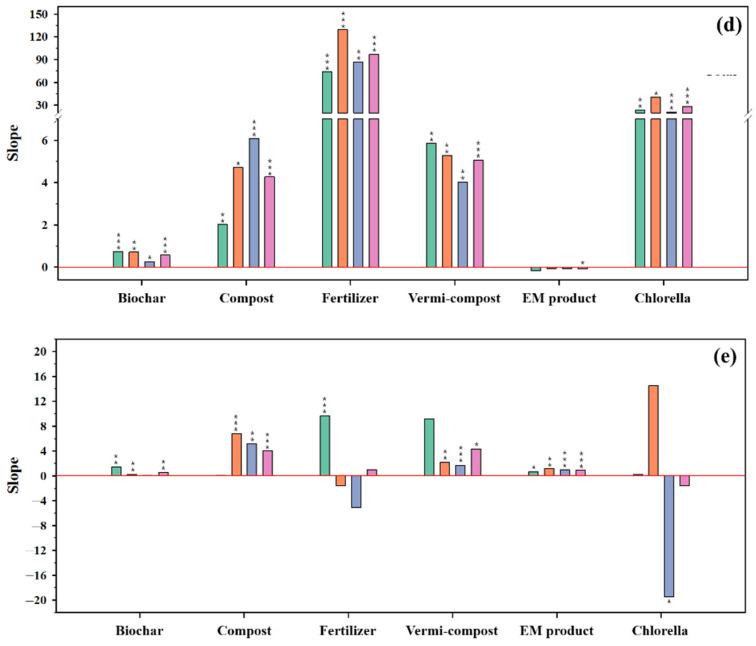
Results of the simple regression analyses according to the treatment periods. The first, second, and third periods’ mean after 2, 6, and 10 weeks. (**a**) Water holding capacity (WHC), (**b**) exchangeable K (Ex. K), (**c**) nitrate-nitrogen (NO3-N), (**d**) available phosphorus (Av. P), and (**e**) urease. * Significance (*p*-value) ≤ 0.05, ** Significance (*p*-value) ≤ 0.01, and *** Significance (*p*-value) ≤ 0.001.

As mentioned in Section 3.2, the properties that deteriorated through the landfarming process were WHC, Ex. K, NO_3_-N, Av. P, and urease, and the results of their simple regression analyses are given in Figure 2. In the case of WHC, peat moss showed the most considerable significance and the largest slope (Figure 2a). Following peat moss, chlorella and compost displayed excellent effectiveness in improving WHC. Ex. K was effectively enhanced by fertilizer, chlorella, zeolite, and biochar (Figure 2b). Fertilizer exhibited the best recovery of NO_3_-N followed by vermicompost, but the latter’s effect decreased with time (Figure 2c). In addition, Av. P was significantly improved by fertilizer, chlorella, vermicompost, and compost (Figure 2d). Except for the EM product, most of the amendments showed an excellent effect for Av. P. The activity of urease was remarkably influenced by chlorella, but its effect was not significant because it showed opposite tendencies according to the period of recovery (Figure 2e). Compost did not show any effect until the second week, but its significance increased afterwards. Even though EM product could supply microorganisms to the soil, its effect seemed to be feeble in activating soil enzymes, such as urease.

**Figure 3:**
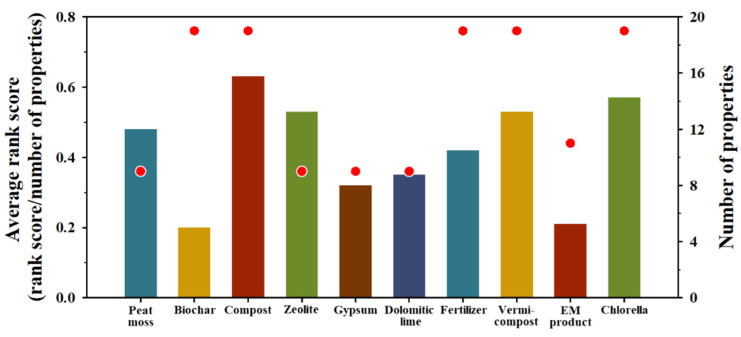
Average rank scores of each amendment calculated based on the results of the simple regression analyses. Refer to Section 3.3 for the detailed explanation. Bar graphs and dots designate the rank score of each material and the number of properties analyzed, respectively.

In order to rehabilitate the health of remediated soil, it is crucial to improve whole properties deteriorated through the landfarming process rather than to revitalize individual properties. Hence, a combination of relevant amendments should be taken into account. To attain this goal, among 10 materials tested, the most relevant ones were determined based on the results of simple regression analyses following the process: (i) extracting the properties of each material with 95% significance; (ii) determining the rank of each material according to the slope values of properties extracted through step (i); (iii) computing the score of each material using the reciprocal of their rank, e.g., if the number of materials for a certain property was 7, then the scores of the seventh and first ranks were calculated as 1 and 7, respectively; (iv) normalizing the scores through dividing each score by total number of materials, e.g., if total number of materials was 7, then the normalized scores of 1and 7 were 0.14 (1/7) and 1.00 (7/7), respectively; (v) calculating the normalized scores for each material; and (vi) determining final scores of each material through dividing their normalized scores by their total number of properties, regardless of the significance considered in step (i). Final scores of each material calculated through the abovementioned process are given in Figure 3. Compost was confirmed as the most effective among the biological materials, and its score was 0.63. The scores of chlorella and vermicompost, the most effective among the biological amendments, were 0.57 and 0.53, respectively. Zeolite showed the highest score among the inorganic agents. The effects of compost, chlorella, and vermicompost were verified to improve deteriorated properties, and they also exhibited the highest-ranking scores. On the contrary, although zeolite was not effective in enhancing degraded properties, it showed a relatively higher score. Meanwhile, biochar and EM materials had the lowest scores among the materials tested, and they seemed to be ineffective in recovering the health of soil remediated by the landfarming process in this study. However, other studies have reported that they are excellent materials in improving soil health [27]. Consequently, compost, chlorella, and vermicompost not only showed excellent effects in improving the properties deteriorated through the landfarming process, but their overall effects were demonstrated when considering all the properties affecting the health of remediated soil.

### 3.4. Correlation Analyses through PCA

The influential materials should improve the deteriorated properties and other properties affecting the health of remediated soil. To scrutinize their overall effect on soil health, the interrelationship between deterioration and other properties during the recovery process was assessed. In particular, the degraded properties and their degree of deterioration are different due to the difference of various factors, such as the duration and operational conditions of the landfarming process, the specificity of the site, and the inherent property of soil, and it is crucial to analyze the interrelationship between deteriorated and the other properties of soils during the restoration period. As mentioned in Section 2.3.2, instead of conventional correlation analyses, correlation analyses using PCA were conducted to enhance the reliability of the results. Table S5 summarizes the PCA results for compost, chlorella, and vermicompost. The number of PCs was determined in the case of their eigenvalues greater than or equal to one (Figure S7). Component loadings were estimated using PC’s eigenvalues, and the component loadings are given in Table S6 according to each soil property. The normalized correlation coefficients between deteriorated and the other properties are presented in Table 4.

In terms of WHC, compost showed higher positive correlation coefficients compared with those of chlorella and vermicompost, and average and median values were estimated as 0.71 and 0.77, respectively (Table 4). The average values of correlation coefficients of Ex. K were 0.57 for compost and 0.54 for chlorella. In the case of NO_3_-N, the correlation coefficients of vermicompost appeared to be higher than those of compost and chlorella, and the average value was 0.57. For Av. P, the average correlation coefficients of compost, vermicompost, and chlorella were 0.67, 0.65, and 0.43, respectively. Finally, compost showed relatively higher correlation coefficients for Ure than other materials. Overall, compost exhibited higher positive correlation coefficients, and in particular, the values for fertility and microbial (soil enzyme) factors were significantly higher. Among deteriorated properties, however, Ex. K showed the lowest correlation with other properties, which might be attributed to the lower content of K in compost (Table 4). In the case of vermicompost, most of the degraded properties were highly correlated with other properties, except for WHC. Similarly, Singh et al. (2017) reported that vermicompost effectively promoted soils’ fertility and microbial factors [81]. The poor correlation of WHC might be caused by a stronger tendency of aggregation of vermicompost, i.e., vermicompost was not well dispersed in the soil, and its effect in improving WHC was diminished, despite its contribution to improving the SOM content (Figure 1a and Figure S5a). In addition, the correlation coefficient of WHC with AS in vermicompost was computed as −0.83. Generally, WHC and AS should be positively correlated with the SOM content, but they appeared to be negatively correlated with each other, although vermicompost seemed to enhance the SOM content; the analytical process of AS likely caused their negative correlation. The AS was measured by weighing the aggregated portion of soil after its dispersion and wet sieving, and the strong aggregation tendency of vermicompost might distort the weight of the aggregated portion of the soil. In terms of chlorella, the correlation of WHC and Ex. K with other properties was positive overall, but the other deteriorated properties—such as NO_3_-N, Av. P, and Ure—showed lower correlations. Chlorella was clearly confirmed to be effective in improving most of the deteriorated properties, except for Ure, but its correlation with other properties was determined to be very low, indicating that chlorella seemed to be effective for individual properties.

## 4. Conclusions

Soil health can be degraded by contamination and through remediation processes, and it should be rehabilitated to extend the purposes of its reuse and/or recycling. Generally, landfarming has been considered an environmentally friendly remediation technology, but the results of this study indicated that some of the soil properties—such as WHC, Ex. K, NO_3_-N, Av. P, and Ure—were related to the soil health deterioration during the landfarming process. The applicability of 10 materials was tested to improve those deteriorated properties. Overall, the statistical results suggested that compost, vermicompost, and chlorella were not only the most effective amendments in promoting the properties degraded during the landfarming process or needed to be improved to meet the criteria for the reuse and recycling of agricultural and landscape purposes, but also their correlation with other properties was generally positive rather than negative. Their effectiveness tended to be increased with increasing dosage, but the smaller amount of about 2.5–5.0% seemed to be sufficient to meet the criteria for reuse and recycling. In addition, the effects of those three materials seemed to be complementary, indicating that the optimal combination of relevant amendments is critical to rehabilitating the health of remediated soils for their reuse and recycling.

## Figures and Tables

**Table 1:** Properties of amendments used.

Properties			Organic Material		Inorganic Material			Fertili-Zer		Organism Material		
			Peat Moss	Biochar	Compost	Zeolite	Gypsum	Dolomitic Lime		Vermi-Compost	Chlorella	EM Product
Physicochemical factor	Texture (%)	Sand	-	-	-	69.10 ± 2.43	72.11 ± 4.51	56.87 ± 2.04	-	-	-	-
		Silt	-	-	-	28.79 ± 2.23	25.34 ± 3.99	38.30 ± 1.93	-	-	-	-
		Clay	-	-	-	2.11 ± 0.18	2.55 ± 0.53	4.84 ± 0.11	-	-	-	-
	pH		3.91 ± 0.04	10.14 ± 0.01	6.98 ± 0.01	6.91± 0.05	5.69 ± 0.01	10.09 ± 0.01	7.16 ± 0.01	6.40 ± 0.00	6.73 ± 0.01	7.52 ± 0.12
	EC (mS/cm)		0.17 ± 0.01	1.43 ± 0.03	4.47 ± 0.15	0.12 ± 0.07	8.55 ± 0.96	2.05 ± 0.02	110.40± 1.70	3.30 ± 0.13	3.51 ± 0.04	6.35 ± 0.16
	Ex-Na (cmolc/kg)		0.27 ± 0.01	0.35 ± 0.02	11.24 ± 1.36	8.46 ± 0.31	2.28 ± 0.12	1.95 ± 0.11	23.56 ± 2.28	5.75 ± 0.34	3.62 ± 0.18	-
	Ex-K (cmolc/kg)		0.27 ± 0.01	13.88 ± 0.82	4.26 ± 0.53	6.44 ± 0.18	4.42 ± 0.12	0.14 ± 0.00	149.36 ± 10.15	4.44 ± 0.25	24.49 ± 0.99	-
	Ex-Mg (cmolc/kg)		3.92 ± 0.06	0.51 ± 0.08	18.04 ± 2.06	9.92 ± 0.23	19.31 ± 0.84	1.62 ± 0.10	0.59 ± 0.01	18.21 ± 0.92	18.21 ± 0.74	-
	Ex-Ca (cmolc/kg)		4.45 ± 0.05	1.01 ± 0.07	41.75 ± 3.53	21.37 ± 0.41	19.43 ± 0.82	112.38 ± 0.58	10.93 ± 0.27	57.88 ± 1.89	6.28 ± 0.26	-
	CEC (cmolc/kg)		-	-	10.83 ± 1.48	44.82 ± 0.75	2.87 ± 0.18	2.50 ± 0.77	8.84 ± 1.22	65.61 ± 3.78	9.67 ± 0.28	-
Fertility factor	SOM (%)		44.06 ± 0.26	11.00 ± 0.18	51.78 ± 0.37	1.79 ± 0.05	12.43 ± 0.26	35.93 ± 0.35	19.21 ± 0.77	47.09 ± 0.20	72.23 ± 1.29	3.36 ± 0.26
	T-N (%)		-	0.20 ± 0.06	1.69 ± 0.17	-	-	-	10.49 ± 0.25	2.35 ± 0.36	8.53 ± 0.42	0.01 ± 0.00
	NO_3_-N (g/kg)		-	0.39 ± 0.03	0.70 ± 0.04	-	-	-	19.82 ± 1.14	3.64 ± 0.08	9.25 ± 0.19	0.15 ± 0.01
	Available P (g/kg)		-	2.66 ± 0.21	5.34 ± 0.03	-	-	-	52.08 ± 4.69	0.69 ± 0.04	7.66 ± 0.21	0.00 ± 0.00

**Table 2:** Properties of each material monitored during the restoration process.

Properties.		Organic Material			Inorganic Material			Fertilizer	Organism Material		
		Peat Moss	Compost	Biochar	Zeolite	Gypsum	Dolomitic Lime		Vermi-compost	Chlorella	EM Product
Physicochemical factor	WHC	○	○	○	○	○	○	○	○	○	
	Soil texture				○	○	○				
	Aggregate stability	○	○	○	○	○	○	○	○	○	
	pH	○	○	○	○	○	○	○	○	○	
	EC	○	○	○	○	○	○	○	○	○	
	CEC	○	○	○	○	○	○	○	○	○	
	Exchangeable cation	○	○	○	○	○	○	○	○	○	
Fertility factor	SOM	○	○	○	○	○	○	○	○	○	○
	T-N		○	○				○	○	○	○
	NO_3_-N		○	○				○	○	○	○
	Available P		○	○				○	○	○	○
Microbial (soil enzyme) factor	β-glucosidase		○	○				○	○	○	○
	N-acetyl-β-glucosaminidase		○	○				○	○	○	○
	Urease		○	○				○	○	○	○
	Alkaline phosphatase		○	○				○	○	○	○
	Acid phosphatase		○	○				○	○	○	○
	Arylsulfatase		○	○				○	○	○	○
	Dehydrogenase		○	○				○	○	○	○

**Table 3:** Comparison of soil properties between non-contaminated soil (NS), contaminated soil (CS), and remediated soil (RS) and the determination of deteriorated properties which should be improved, based on the criteria of soil quality standards and the properties of NS.

Soil Property		NS	CS	RS	Changes in RS Based on NS	Changes in RS Based on CS	Classification on Recovery of Soil Quality	Criteria of Soil Quality Standards
Physicochemical factor	WHC (%)	26.3 ± 0.21	25.2 ± 0.18	24.8 ± 0.28	−1.5	−0.4	O ^1^	NS
	Texture	Loamy sand	Sandy loam	Sandy loam	-	-	X ^2^	NS
	Aggregate stability (%)	34.5 ± 2.1	10.2 ± 0.1	44.2 ± 1.4	9.7	34.0	X	NS
	pH	5.8 ± 0.1	7.3 ± 0.0	6.0 ± 0.1	0.2	−1.3	X	5.5–7.0 *, 4.5−8.0 **
	EC (µS/cm)	16.2 ± 0.3	70.4 ± 0.8	70.2 ± 1.3	54	−0.2	X	NS, <1500 **
	Ex. Na (mg/kg)	0.06 ± 0.00	0.05 ± 0.00	0.14 ± 0.01	0.08	0.09	X	NS
	Ex. K (mg/kg)	0.22 ± 0.01	0.20 ± 0.01	0.30 ± 0.02	0.08	0.1	O	0.25–0.80 *, >0.6 **
	Ex. Ca (mg/kg)	5.05 ± 0.35	9.23 ± 1.13	5.41 ± 0.69	0.36	−3.82	X	5.0–7.0 *, >2.5 **
	Ex. Mg (mg/kg)	2.80 ± 0.11	3.19 ± 0.24	1.62 ± 0.08	−1.18	−1.57	X	1.2–2.0 *, >0.6 **
	CEC (cmolc/kg)	11.5 ± 0.66	11.3 ± 0.43	11.6 ± 0.56	0.1	0.3	X	NS, >6 **
Fertility factor	SOM (g/kg)	27.4 ± 0.6	28.0 ± 0.2	35.0 ± 0.4	7.6	7.0	X	20–30 *, >30 **
	T-N (mg/kg)	610 ± 23.9	149 ± 9.3	638 ± 36.6	28	489	X	NS, >600 **
	NO_3_-N (mg/kg)	2.47 ± 0.16	1.95 ± 0.13	1.74 ± 0.08	−0.73	−0.21	O	NS
	Available P (mg/kg)	0.13 ± 0.01	4.32 ± 0.15	16.02 ± 0.39	15.89	11.7	O	35–218 *, >43.7 **
Microbial (soil enzyme) factor	β-glucosidase (μg-PNP/g-soil-hr)	4.3 ± 0.1	18.0 ± 0.6	35.3 ± 1.07	31	17.3	X	NS
	N-acetyl-β-glucosaminidase (μg-PNP/g-soil-hr)	9.8 ± 0.3	13.3 ± 0.9	20.0 ± 0.7	10.2	6.7	X	NS
	Urease (NH_4_^+^produced/g-DW soil∙hr)	35.4 ± 1.4	23.2 ± 1.1	26.3 ± 0.9	−9.1	3.1	O	NS
	Acid phosphatase (μg-PNP/g-soil-hr)	14.1 ± 0.3	33.4 ± 1.5	35.6 ± 1.0	21.5	2.2	X	NS
	Alkaline phosphatase (μg-PNP/g-soil-hr)	70.3 ± 2.2	63.8 ± 3.9	70.8 ± 2.4	0.5	7.0	X	NS
	Arylsulfatase (μg-PNP/g-soil-hr)	1.9 ±0.2	4.2 ± 0.2	22.8 ± 0.9	20.9	18.6	X	NS
	Dehydrogenase (µgTPF/g-DW Soil)	0.8 ± 0.0	2.0 ± 0.1	4.0 ± 0.3	3.2	2.0	X	NS

^1^ The property needed to be recovered or improved, ^2^ The property needed not to be recovered or improved, * Criteria for agricultural uses (refer to Table S3), ** Criteria for landscape (forest field) uses (refer to Table S4)

**Table 4:** Normalized correlation coefficients between deteriorated and other soil properties for three most effective amendments. ** Significance (*p*-value) ≤ 0.01, * Significance (*p*-value) ≤ 0.05.

		Compost	Vermi-Compost	Chlorella	Compost	Vermi-Compost	Chlorella	Compost	Vermi-Compost	Chlorella	Compost	Vermi-Compost	Chlorella	Compost	Vermi-Compost	Chlorella
		WHC			Ex-K			NO_3_-N			Av. P			Ure		
Physicochemical factor	WHC	0.89 **	0.91 **	0.87 **	0.34	*−*0.30	0.90 **	0.39	*−*0.32	0.17	0.78 **	0.18	0.68 **	0.62 *	*−*0.68 **	−0.04
	AS	0.53 *	*−*0.83 **	0.12	*−*0.31	0.51	0.14	*−*0.14	0.54 *	0.49	0.58 *	0.18	*−*0.19	0.62 *	0.79 **	0.08
	pH	0.64 *	0.15	0.15	0.63 *	0.12	0.09	0.48	0.18	0.00	0.44	0.45	*−*0.35	0.22	0.04	−0.27
	EC	0.88 **	*–*0.18	0.82 **	0.54 *	0.76 **	0.79 **	0.21	0.76 *	*–*0.16	0.59 *	0.81 *	0.76 **	0.38	0.64 *	0.05
	Ex-Na	0.80 **	*–*0.28	0.89 **	0.58 *	0.93 **	0.92 **	0.62 *	0.89 **	0.15	0.67 *	0.83 **	0.73 **	0.46	0.79 **	−0.04
	Ex-K	0.34	*–*0.30	0.90 **	0.93 **	0.94 **	0.98 **	0.25	0.88 **	0.34	*–*0.07	0.76 **	0.69 **	*−*0.34	0.80 **	−0.21
	Ex-Mg	0.70 **	0.02	0.88 **	0.44	0.84 **	0.98 **	0.81 **	0.80 **	0.38	0.72 **	0.89 **	0.68 **	0.55 *	0.58 *	−0.21
	Ex-Ca	0.77 **	0.11	0.17	0.33	0.74 **	0.33	0.77 **	0.71 **	0.74 **	0.83 **	0.90 **	*−*0.14	0.68 **	0.47	−0.45
	CEC	0.25	*–*0.51 *	0.56 *	0.13	0.26	0.61 *	0.86 **	0.33	*–*0.06	0.50	0.21	0.84 **	0.44	0.47	0.09
Fertility factor	SOM	0.85 **	0.54 *	0.72 **	0.21	0.21	0.87 **	0.65**	0.22	0.40	0.91 **	0.67 **	0.61 *	0.79 **	*−*0.11	−0.41
	T-N	0.92 **	0.46	0.79 **	0.24	0.50	0.75 **	0.43	0.47	*−*0.20	0.87 **	0.84 **	0.82 **	0.74 **	0.12	0.21
	NO_3_-N	0.39	*−*0.32	0.17	0.25	0.88 **	0.34	0.83 **	0.84 **	0.74 **	0.55 *	0.72 **	*−*0.03	0.45	0.78 **	−0.36
	Av. P	0.78 **	0.18	0.68 **	*−*0.07	0.76 **	0.69 **	0.55*	0.72 **	*−*0.03	0.95 **	0.92 **	0.83 **	0.91 **	0.45	0.21
Microbial soil enzyme) factor	BG	0.74 **	*−*0.20	0.68 **	0.48	0.59 *	0.55 *	*−*0.09	0.61 *	*−*0.34	0.38	0.66 **	0.53 *	0.20	0.53 *	0.27
	NAG	0.88 **	*−*0.11	0.79	0.58 *	0.75 **	0.71 **	0.31	0.75 **	*−*0.15	0.62*	0.84 **	0.54 *	0.40	0.60 *	0.09
	URE	0.62 *	*−*0.68 **	*−*0.04	*−*0.34	0.80 **	*−*0.21	0.45	0.78 **	*−*0.36	0.91 **	0.45	0.21	0.94 **	0.91 **	0.88
	ACP	0.82 **	*−*0.37	0.38	*−*0.02	0.48	0.17	0.46	0.52 *	*−*0.53	0.92 **	0.49	0.29	0.86 **	0.54 *	0.52 *
	ALP	0.91 **	0.14	0.38	0.49	0.67 **	0.23	0.57 *	0.65 **	*−*0.45	0.78 **	0.89 **	0.16	0.58	0.41	0.14
	ARS	0.87 **	*−*0.44	0.62 *	0.07	0.92 **	0.74 **	0.52 *	0.89 **	0.36	0.94 **	0.73 **	0.71 **	0.85 **	0.87 **	−0.10
	DHA	0.73 **	0.26	0.64 *	0.40	0.49	0.61*	0.74 **	0.49	*−*0.22	0.75 **	0.81 **	0.72 **	0.58 *	0.23	0.15
Min		0.25	*−*0.83	*−*0.04	*−*0.34	*−*0.30	*−*0.21	*−*0.14	*−*0.32	*−*0.53	*−*0.07	0.18	*−*0.35	*−*0.34	*−*0.68	*−*0.45
Max		0.92	0.54	0.90	0.63	0.93	0.98	0.86	0.89	0.74	0.94	0.90	0.84	0.91	0.87	0.52
Average		0.71	*−*0.07	0.56	0.26	0.57	0.54	0.46	0.57	0.03	0.67	0.65	0.43	0.53	0.44	*−*0.01
Median		0.77	−0.15	0.66	0.33	0.67	0.61	0.48	0.65	−0.03	0.72	0.73	0.61	0.58	0.53	0.05
S.D.		0.20	0.43	0.30	0.29	0.32	0.33	0.27	0.30	0.35	0.25	0.25	0.38	0.29	0.38	0.25

## Data Availability

The data presented in this study are available on request from the corresponding author.

## Supplementary Materials

The following supporting information can be downloaded at: https://www.mdpi.com/article/10.3390/toxics10030147/s1, Figure S1: Methods and examples of simple regression analyses. (a) Linear relationship between independent and dependent variables on one variable and (b) examples of simple regression analysis data on individual amendments used in this study, Figure S2: Soil textures of non-contaminated (NS), contaminated (CS), and remediated (RS) soils, Figure S3: Changes in physicochemical factor according to the dosage of each amendment and the duration of treatment. (a) aggregate stability (AS), (b) pH, (c) electrical conductivity (EC), (d) exchangeable Na (Ex. Na), (e) exchangeable Ca (Ex. Ca), (f) exchangeable Mg (Ex. Mg), and (g) cation exchange capacity (CEC). The 1st, 2nd, and 3rd periods mean after 2, 6, 10 weeks of treatment. The red dotted lines are the upper limit of the criteria of soil quality standard for agricultural and landscape (forest field) uses. Each result was normalized by subtracting the values of remediated soil (RS) from the values of restored soil, Figure S4: Changes in soil textures according to the dosage of each amendment and the duration of treatment. (a) zeolite after the 1st period (2 weeks), (b) zeolite after the 2nd period (6 weeks), (c) zeolite after the 3rd period (10 weeks), (d) gypsum after the 1st period (2 weeks), (e) gypsum after the 2nd period (6 weeks), (f) gypsum after the 3rd period (10 weeks), (g) dolomitic lime after the 1st period (2 weeks), (h) dolomitic lime after the 2nd period (6 weeks), and (i) dolomitic lime after the 3rd period (10 weeks), Figure S5: Changes in fertility factor according to the dosage of each amendment and the duration of treatment. (a) soil organic matter (SOM) and (b) total nitrogen (T-N). The 1st, 2nd, and 3rd periods mean after 2, 6, 10 weeks of treatment. Each result was normalized by subtracting the values of remediated soil (RS) from the values of restored soil, Figure S6: Changes in microbial (soil enzyme activity) factor according to the dosage of each amendment and the duration of treatment. (a) β-glucosidase (BG), (b) N-acetyl-β-glucosaminidase (NAG), (c) acid phosphatase (ACP), (d) alkaline phosphatase (ALP), (e) arylsulfatase (ARS), and (f) dehydrogenase (DHA). The 1st, 2nd, and 3rd periods mean after 2, 6, 10 weeks of treatment. Each result was normalized by subtracting the values of remediated soil (RS) from the values of restored soil, Figure S7: Eigenvalues as a function of component numbers for each amendment obtained from the principal component analyses (PCA). (a) compost, (b) vermicompost, and (c) chlorella, Table S1: Details and corresponding references of analytical methods of soil properties, Table S2: Characteristics of materials used in experiments, Table S3: The criteria of soil quality standards for agricultural uses, Table S4: The criteria of soil quality standards for landscape (forest field) uses, Table S5: Results of principal component analyses (PCA) for three most effective amendments, Table S6: Component loadings on three most effective amendments according to each soil property.
