# Supplementary material for: Revitalization of Total Petroleum Hydrocarbon Contaminated Soil Remediated by Landfarming
Source: Toxics. 2022 Mar 19;10(3):147. doi: 10.3390/toxics10030147 (PMC8951262; doi:10.3390/toxics10030147)
Supplement: Supplementary file 1 [file toxics-10-00147-s001.zip › toxics-1622080-supplementary.pdf]

toxics

MDPI

Revitalization of Total Petroleum Hydrocarbon Contaminated Soil Remediated by Landfarming

Woo-Chun Lee, Jong-Hwan Lee, Sang-Hun Lee, Sang-Woo Lee, Ji-Hoon Jeon, Sang-Hwan Lee, and Soon-Oh Kim

Table S1. Details and corresponding references of analytical methods of soil properties.

<table>
<tr><th></th><th>Type and property</th><th>Analytical method (reference) and instrument</th></tr>
<tr><td>Contaminant</td><td>TPH</td><td>ES 07552.1c TPH-gas chromatography (NIER, 2018) [41]
Gas Chromatograph (GC-2010 Plus, Shimadzu, Japan)</td></tr>
<tr><td rowspan="8">Physicochemical factor</td><td>WHC</td><td>Gravity drainage (OECD, 2008) [42]</td></tr>
<tr><td>Soil texture</td><td>KS F 2032 (KATS, 2017) [43]
Soil physical property analysis (NASS, 2017) [44]
Particle size analyzer (1090LD Shape Analyzer, CILAS, France)</td></tr>
<tr><td>Aggregate stability</td><td>Kemper and Rosenau, 1986 [45]</td></tr>
<tr><td>pH</td><td rowspan="2">1 : 5 H_2O (NAAS, 2010) ([46], pp. 23–29)
Benchtop multimeter (Orion Star A215, Thermo Scientific Orion, USA)</td></tr>
<tr><td>EC</td></tr>
<tr><td>Exchangeable cation</td><td>1M ammonium acetate (US EPA, 1986) ([47], pp. 1–9)
ICP-OES (CAP PRO X, Thermo Scientific™, USA)</td></tr>
<tr><td>CEC</td><td>1M sodium acetate (US EPA, 1986) ([47], pp. 1–4)
ICP-OES (CAP PRO X, Thermo Scientific™, USA)</td></tr>
<tr><td rowspan="4">Fertility factor</td><td>SOM</td><td>Walkely & black (NAAS, 2010) ([46], pp. 51–60)
UV-Vis spectrophotometer (UV-1900i, Shimadzu, Japan)</td></tr>
<tr><td>Total Nitrogen</td><td>Kjeldahl (NAAS, 2010) ([46], pp. 63–68)</td></tr>
<tr><td>NO_3-N</td><td>Kjeldahl distillation (NAAS, 2010) ([46], pp. 75–84)</td></tr>
<tr><td>Available P</td><td>Bray No.1 (NAAS, 2010) +
([46], pp. 95–124)
UV-Vis spectrophotometer (UV-1900i, Shimadzu, Japan)</td></tr>
<tr><td rowspan="7">Microbial (soil enzyme) factor</td><td>β-glucosidase</td><td rowspan="2">Stott, 2019 [48]
UV-Vis spectrophotometer (UV-1900i, Shimadzu, Japan)</td></tr>
<tr><td>N-acetyl-β-glucosaminidase</td></tr>
<tr><td>Urease</td><td>Kandeler and Gerber, 1988 [49]
UV-Vis spectrophotometer (UV-1900i, Shimadzu, Japan)</td></tr>
<tr><td>Acid phosphatase</td><td rowspan="3">Stott, 2019 [48]
UV-Vis spectrophotometer (UV-1900i, Shimadzu, Japan)</td></tr>
<tr><td>Alkaline phosphatase</td></tr>
<tr><td>Arylsulfatase</td></tr>
<tr><td>Dehydrogenase</td><td>Pepper and Gerba, 2004 [50]
UV-Vis spectrophotometer (UV-1900i, Shimadzu, Japan)</td></tr>
</table>

TPH: total petroleum hydrocarbon, WHC: water holding capacity, EC: electrical conductivity, CEC: cation exchange capacity, SOM: soil organic matter.

41. *ES 07552.1c*; TPH-Gas Chromatography. National Institute of Environmental Reserch: Incheon, Korea, 2018.
42. OECD. *OECD Guideline for the Testing of Chemicals No. 314-Simulation Tests to Assess the Biodegradability of Chemicals Discharged in Wastewater*; Organisation for Economic Cooperation and Development (OECD): Paris, France, 2008.
43. *KS F 2032*; Standard Test Method for Particle Size Distribution of Soils. Korean Agency for Technology and Standards: Eumseong, Korea, 2017; pp. 1–18.
44. Yun, Y.K.; *Investigation and Analysis Methods for Physical Properties of Soil*; Rural Development Administration National Academy of Agricultural Science: Seoul, Korea, 2017; pp. 13–31.
45. Kemper, W.D.; Rosenau, R.C. Aggregate stability and size distribution. In *Methods of Soil Analysis, Part 1: Physical and Mineralogical Methods*; Klute, A., Ed.; Madison, Wisconsin, USA, 1986; pp. 425–442.

46. National Academy of Agricultural Science. *Methods of Soil Chemical Analysis*; Rural Development Administration: Wanju, Korea, 2010; pp. 23–29.
47. *SW-846; Test Method 9080: Cation-Exchange Capacity of Soils (Ammonium Acetate). United States Environmental Protection Agency: Washington, D.C., USA, 1986; pp. 1–9*.
48. Stott, D.E. *Recommended Soil Health Indicators and Associated Laboratory Procedures*; Soil Health Technical Note No. 450–03; U.S. Department of Agriculture, Natural Resources Conservation Service: Washington, D.C., USA, 2019.
49. Kandeler, E.; Gerber, H. Short-term assay of soil urease activity using colorimetric determination of ammonium. *Biol. Fertil. Soils* **1988**, *6*, 68–72.
50. Pepper, I.L.; Gerba, C.P. *Environmental Microbiology: A Laboratory Manual*, 2nd ed.; Elsevier Academic Press: Cambridge, MA, USA, 2004.

Table S2. Characteristics of materials used in experiments.

Material		Picture	Effect	Specification
Organic material	Peat moss	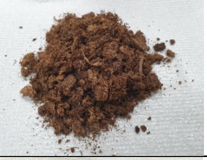	Enhancing water retention, drainage, and breathability	pH: 3.5–4.5, Laflora white peat moss (directly imported from North Europe)
	Compost	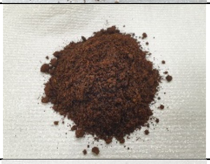	Improving fertility and nourishment	Pig manure 40%, chicken manure 15%, sawdust 27%, natural humus 10%, zeolite 5%, and soil microbial agent 3%
	Biochar	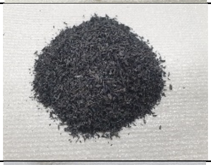	Controlling moisture and oxygen contents (containing oxygen up to 30% of its own volume)	Direct carbonization method (complete carbonization), 100% rice husk
Inorganic material	Zeolite	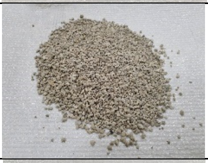	Preventing acidification, improving hydration and water retention, supplying deficient ingredients, and buffering excess fertilization	100% natural zeolite (porous feldspar mineral)
	Gypsum	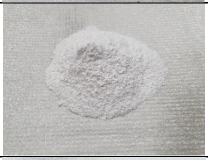	Promoting soil water holding capacity and agglomerate stability	Calcium sulfate (99% or more)
	Dolomitic lime	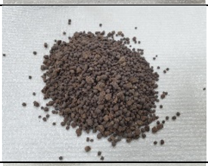	Supplying nutrients, improving soil structure, and promoting microbial activity	Soluble magnesia 14%, alkali content 51%, amino acids, organic matter, and trace nutrients
Fertilizer		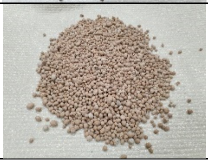	Supplying nutrients and increasing exchangeable cations and fertility	Nitrogen (N) 11%, phosphoric acid (P) 7%, potassium (K) 10%, goto (MgO) 2%, boron 1.2%
Organism material	Vermi-compost	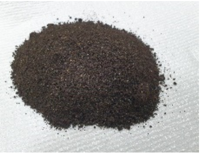	Promoting soil agglomeration, improving physical properties (drainability, breathability, water retention), and supplying inorganic nutrients necessary for plant growth	Manufacturing fecal soil using organic matter as food for earthworms, Using natural circulation farming methods
	Chlorella	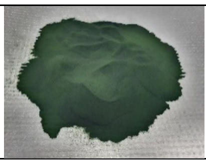	Supplying nutrients, increasing soil enzyme activity, promoting plant growth, and preventing pests and diseases	Chlorella raw powder (99%, containing beta-carotene, magnesium, minerals, potassium), vitamin C 1%
	EM product	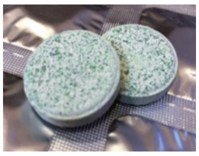	Promoting soil enzyme activity, increasing the absorption rate of nutrients by plants, and preventing pests and diseases	Including 10 types of microorganisms (Vacillus Laterosporus, Bacillus Megaterium, Paenilbacillus Polymyxa, Bacillus Licheniformis, Bacillus Punilus, Bacillus Subtills, Trichoderma Virde, Trichoderma Harzianum, Trichoderma Polysporum, Trchoderma Koningii)

Table S3. The criteria of soil quality standards for agricultural uses.

Properties	Unit	Agriculture	Green house	Rice paddy	Orchard	This study
pH	-	5.5–6.5	6.0–7.0	6.0–6.5	6.0–6.5	5.5–7.0
EC	μS/cm	≤ 2,000	≤ 2,000	≤ 2,000	≤ 2,000	≤ 2,000
Ex. K	cmol/kg	0.25–0.30	0.70–0.80	0.50–0.60	0.30–0.60	0.25–0.80
Ex. Ca	cmol/kg	5.0–6.0	5.0–7.0	5.0–6.0	5.0–6.05	5.0–7.0
Ex. Mg	cmol/kg	1.5–2.0	1.5–2.0	1.5–2.0	1.2–2.0	1.2–2.0
SOM	g/kg	25–30	20–30	20–30	25–30	20–30
Av. P	mg/kg	34.9–52.4	152.8–218.3	152.8–218.3	87.3–131.0	34.9–218.3

Cited by NAS, Korean soil information system, 2021 (http://soil.rda.go.kr/). (Accessed on 28 January 2022)

Table S4. The criteria of soil quality standards for landscape (forest field) uses.

Properties	Unit	Field soil (landscaping soil)				This study
		High	Medium	Low	Poor	
pH		6.0–6.5	5.5–6.0<br>6.5–7.0	4.5–5.5<br>7.0–8.0	< 4.5<br>> 8.0	4.5–8.0
EC	μS/cm	< 200	200–1,000	1,000–2,000	> 2,000	≤2,000
CEC	cmol/kg	> 20	20–6	< 6	-	> 6
Ex. K	cmol/kg	> 3.0	3.0–0.6	< 0.6	-	> 0.6
Ex. Ca	cmol/kg	> 5.0	5.0–2.5	< 2.5	-	> 2.5
Ex. Mg	cmol/kg	> 3.0	3.0–0.6	< 0.6	-	> 0.6
SOM	g/kg	> 50	30–50	< 30	-	> 30
T-N	mg/kg	> 1200	600–1200	< 600	-	> 600
Av. P	mg/kg	> 87.3	43.7–87.3	< 43.7	-	> 43.7

Cited by KILA, the Korean institute of landscape architecture (2002), Standard for landscaping soil (based on planting).

Table S5. Results of principal component analyses (PCA) for three most effective amendments.

PC	Compost			Vermicompost			Chlorella		
	Eigenvalue	Proportion (%)	Cumulative (%)	Eigenvalue	Proportion (%)	Cumulative (%)	Eigenvalue	Proportion (%)	Cumulative (%)
1	13.01	65.03	65.03	12.57	62.87	62.87	9.64	48.22	48.22
2	2.80	14.00	79.03	3.79	18.92	81.79	4.13	20.66	68.88
3	2.53	12.63	91.65	2.08	10.37	92.17	2.65	13.26	82.14
4	0.52	2.59	94.24	0.53	2.64	94.80	1.08	5.40	87.54
5	0.44	2.21	96.45	0.40	1.97	96.78	0.84	4.18	91.71
6	0.32	1.60	98.04	0.22	1.10	97.87	0.51	2.53	94.24
7	0.17	0.83	98.87	0.18	0.90	98.77	0.42	2.09	96.33
8	0.10	0.49	99.36	0.13	0.63	99.40	0.30	1.52	97.85
9	0.06	0.28	99.63	0.05	0.27	99.67	0.17	0.87	98.72
10	0.03	0.13	99.77	0.03	0.14	99.81	0.11	0.53	99.25
11	0.02	0.11	99.88	0.02	0.09	99.90	0.09	0.46	99.70
12	0.01	0.07	99.94	0.01	0.05	99.96	0.04	0.20	99.90
13	0.01	0.04	99.99	0.01	0.04	100.00	0.02	0.08	99.98
14	0.00	0.02	100.00	0.00	0.00	100.00	0.00	0.02	100.00
15	0.00	0.00	100.00	0.00	0.00	100.00	0.00	0.00	100.00
16	0.00	0.00	100.00	0.00	0.00	100.00	0.00	0.00	100.00
17	0.00	0.00	100.00	0.00	0.00	100.00	0.00	0.00	100.00
18	0.00	0.00	100.00	0.00	0.00	100.00	0.00	0.00	100.00
19	0.00	0.00	100.00	0.00	0.00	100.00	0.00	0.00	100.00
20	0.00	0.00	100.00	0.00	0.00	100.00	0.00	0.00	100.00

Table S6. Component loadings on three most effective amendments according to each soil property.

	Compo-nent	Compost			Vermicompost			Chlorella			
		PC 1	PC 2	PC 3	PC 1	PC 2	PC 3	PC 1	PC 2	PC 3	PC 4
Physico-chemical factor	WHC	0.910	0.250	−0.013	−0.144	−0.924	−0.190	0.898	0.132	0.206	0.073
	AS	0.438	0.509	−0.592	0.543	0.744	0.321	−0.083	0.256	0.592	0.546
	pH	0.702	0.008	0.421	0.622	−0.384	0.611	0.030	−0.276	0.856	−0.228
	EC	0.831	0.493	0.214	0.975	0.027	0.085	0.949	−0.159	−0.025	−0.111
	Ex-Na	0.902	−0.053	0.298	0.928	0.210	−0.263	0.938	0.130	0.143	0.043
	Ex-K	0.356	0.112	0.890	0.809	0.286	−0.447	0.898	0.369	0.194	−0.001
	Ex-Mg	0.871	−0.382	0.194	0.875	−0.085	−0.359	0.873	0.410	0.202	0.040
	Ex-Ca	0.929	−0.302	0.038	0.918	−0.234	−0.141	−0.035	0.735	0.473	0.105
	CEC	0.499	−0.829	0.048	0.573	0.330	0.667	0.739	0.176	−0.615	−0.052
Fertility facto	SOM	0.965	−0.118	−0.134	0.622	−0.719	0.204	0.730	0.571	−0.004	−0.206
	T-N	0.950	0.198	−0.139	0.733	−0.578	−0.158	0.945	−0.188	−0.166	0.019
	NO_3-N	0.611	−0.661	0.122	0.799	0.294	−0.339	−0.007	0.780	0.303	0.194
	Av. P	0.875	−0.103	−0.421	0.868	−0.261	−0.306	0.819	0.073	−0.370	0.144
Microbial factor	BG	0.615	0.738	0.205	0.868	0.025	0.273	0.781	−0.517	0.215	0.035
	NAG	0.870	0.381	0.252	0.985	−0.046	0.074	0.846	−0.319	0.348	−0.011
	URE	0.709	−0.140	−0.647	0.652	0.668	−0.184	0.046	−0.522	−0.297	0.716
	ACP	0.876	0.049	−0.383	0.786	0.175	0.470	0.479	−0.805	0.204	0.190
	ALP	0.975	0.076	0.156	0.935	−0.304	0.011	0.450	−0.671	0.353	−0.174
	ARS	0.939	0.030	−0.297	0.877	0.385	−0.224	0.664	0.560	−0.301	0.095
	DHA	0.882	−0.276	0.130	0.862	−0.454	0.179	0.797	−0.165	−0.241	−0.067

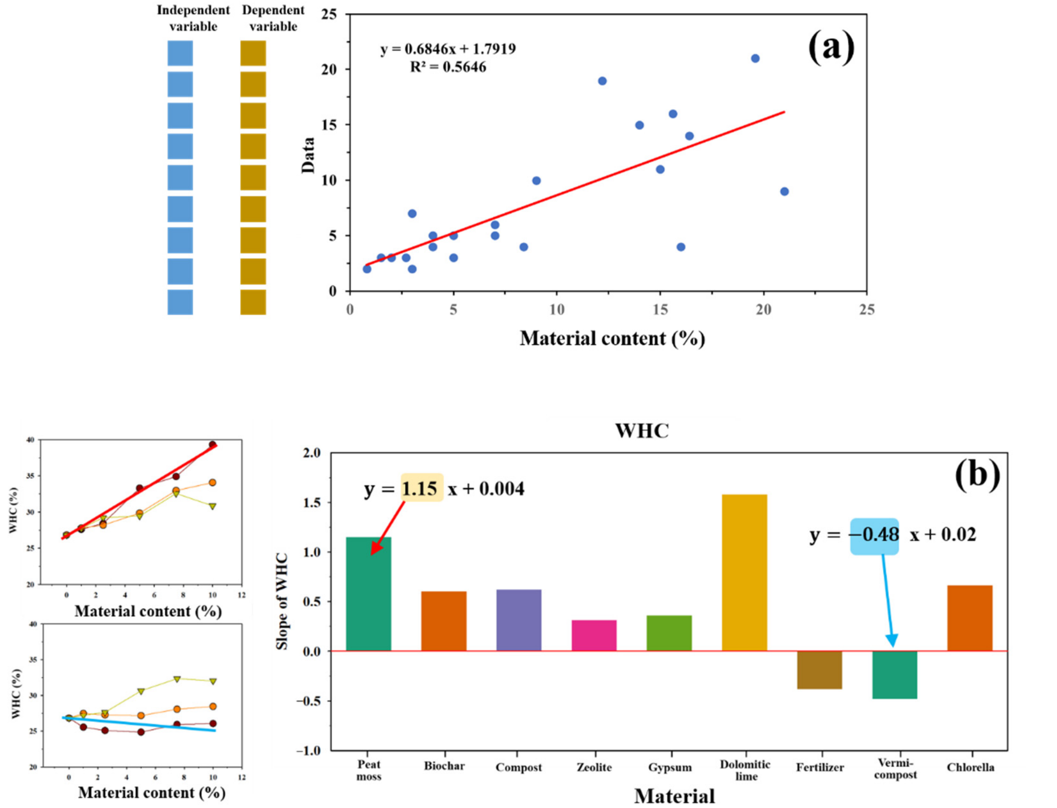

Figure S1. Methods and examples of simple regression analyses. (**a**) Linear relationship between independent and dependent variables on one variable and (**b**) examples of simple regression analysis data on individual amendments used in this study.

1. Regression equations:

$$\boldsymbol{y_i = \beta_0 + \beta_1 x_i + \delta_i \cdot \mathbf{S.t} + \epsilon_i}$$

$\boldsymbol{x_i}$: Dosage of each material

$\boldsymbol{y_i}$: Soil properties (analyzed data)

S.t: Treatment period

$\boldsymbol{\beta_1, \ \delta_i}$: Slopes of the regression equation (regression coefficients)

$\boldsymbol{\epsilon_i}$: Residuals

2. Methodology

(1) Checking the significance of the slopes

If p-value < 0.05, then significant, indicating that corresponding materials and treatment periods are likely effective.

(2) Comparing the slopes of each material

If the larger the slopes of certain material, then it is effective

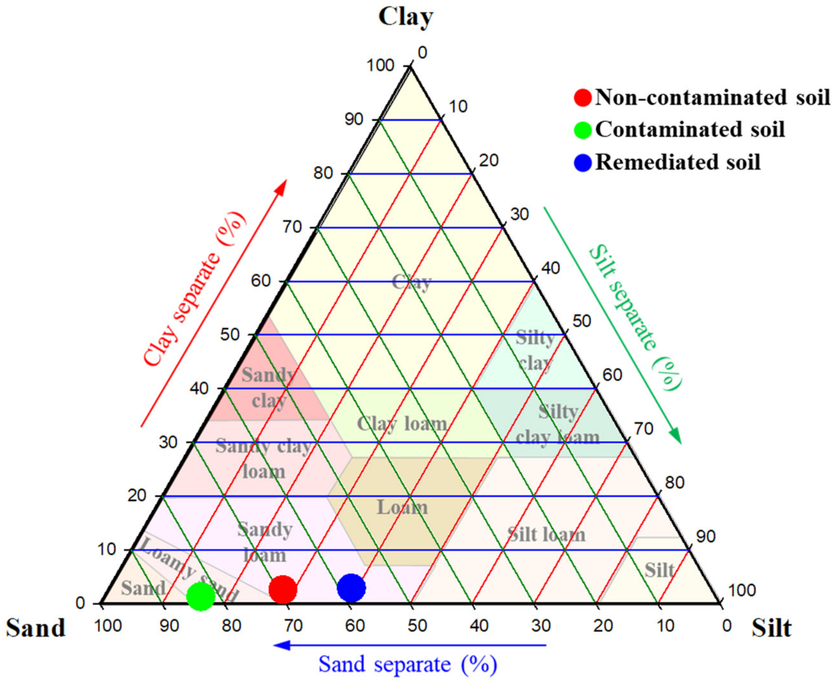

Figure S2. Soil textures of non-contaminated (NS), contaminated (CS), and remediated (RS) soils.

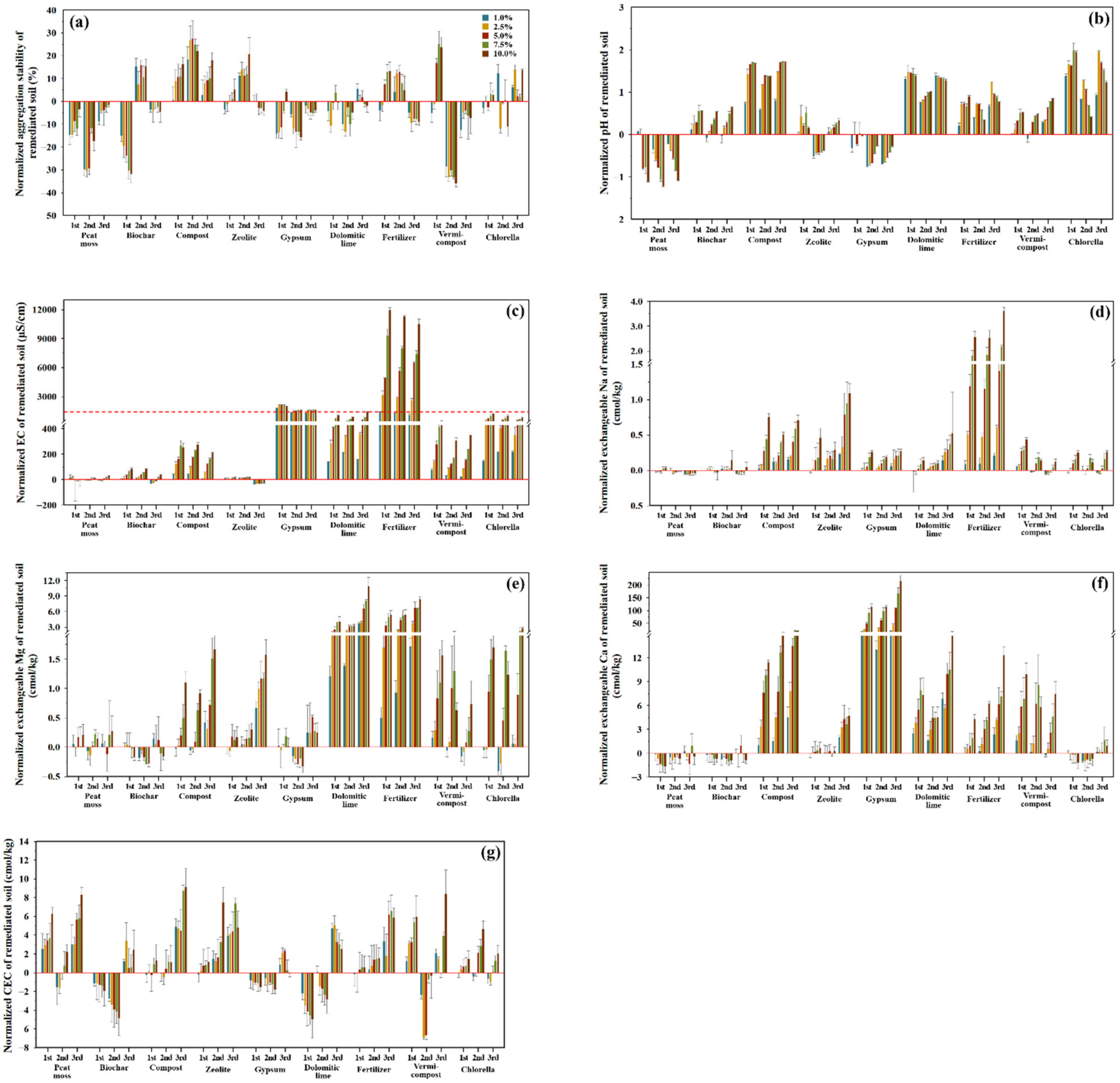

Figure S3. Changes in physicochemical factor according to the dosage of each amendment and the duration of treatment. (**a**) aggregate stability (AS), (**b**) pH, (**c**) electrical conductivity (EC), (**d**) exchangeable Na (Ex. Na), (**e**) exchangeable Ca (Ex. Ca), (**f**) exchangeable Mg (Ex. Mg), and (**g**) cation exchange capacity (CEC). The 1st, 2nd, and 3rd periods mean after 2, 6, 10 weeks of treatment. The red dotted lines are the upper limit of the criteria of soil quality standard for agricultural and landscape (forest field) uses. Each result was normalized by subtracting the values of remediated soil (RS) from the values of restored soil.

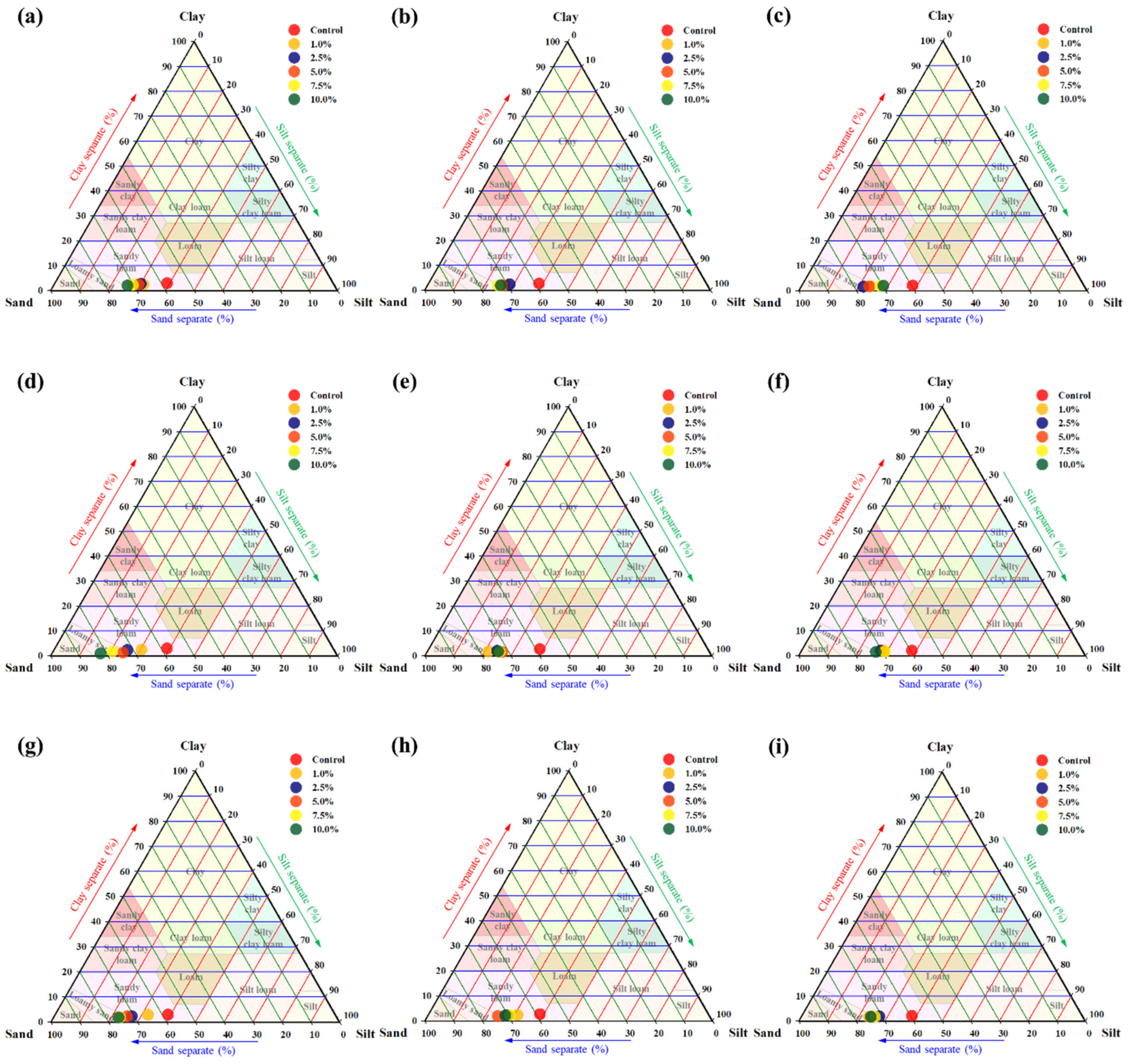

Figure S4. Changes in soil textures according to the dosage of each amendment and the duration of treatment. (**a**) zeolite after the 1st period (2 weeks), (**b**) zeolite after the 2nd period (6 weeks), (**c**) zeolite after the 3rd period (10 weeks), (**d**) gypsum after the 1st period (2 weeks), (**e**) gypsum after the 2nd period (6 weeks), (**f**) gypsum after the 3rd period (10 weeks), (**g**) dolomitic lime after the 1st period (2 weeks), (**h**) dolomitic lime after the 2nd period (6 weeks), and (**i**) dolomitic lime after the 3rd period (10 weeks).

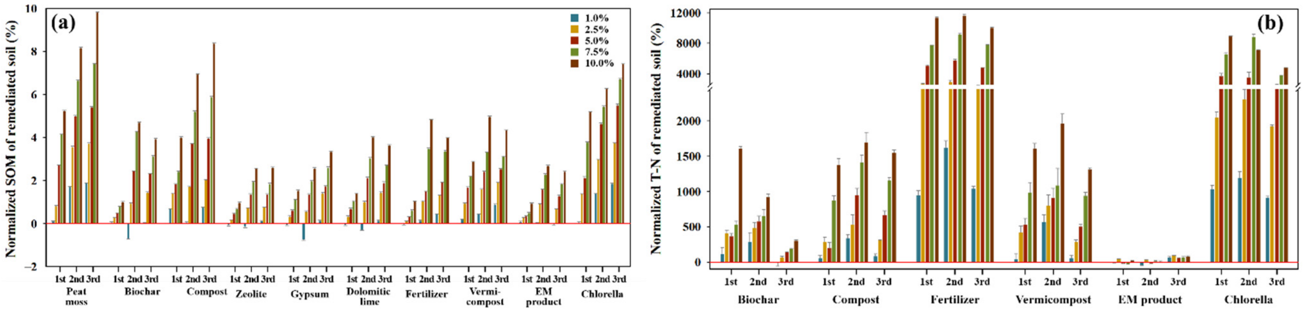

Figure S5. Changes in fertility factor according to the dosage of each amendment and the duration of treatment. (**a**) soil organic matter (SOM) and (**b**) total nitrogen (T-N). The 1st, 2nd, and 3rd periods mean after 2, 6, 10 weeks of treatment. Each result was normalized by subtracting the values of remediated soil (RS) from the values of restored soil.

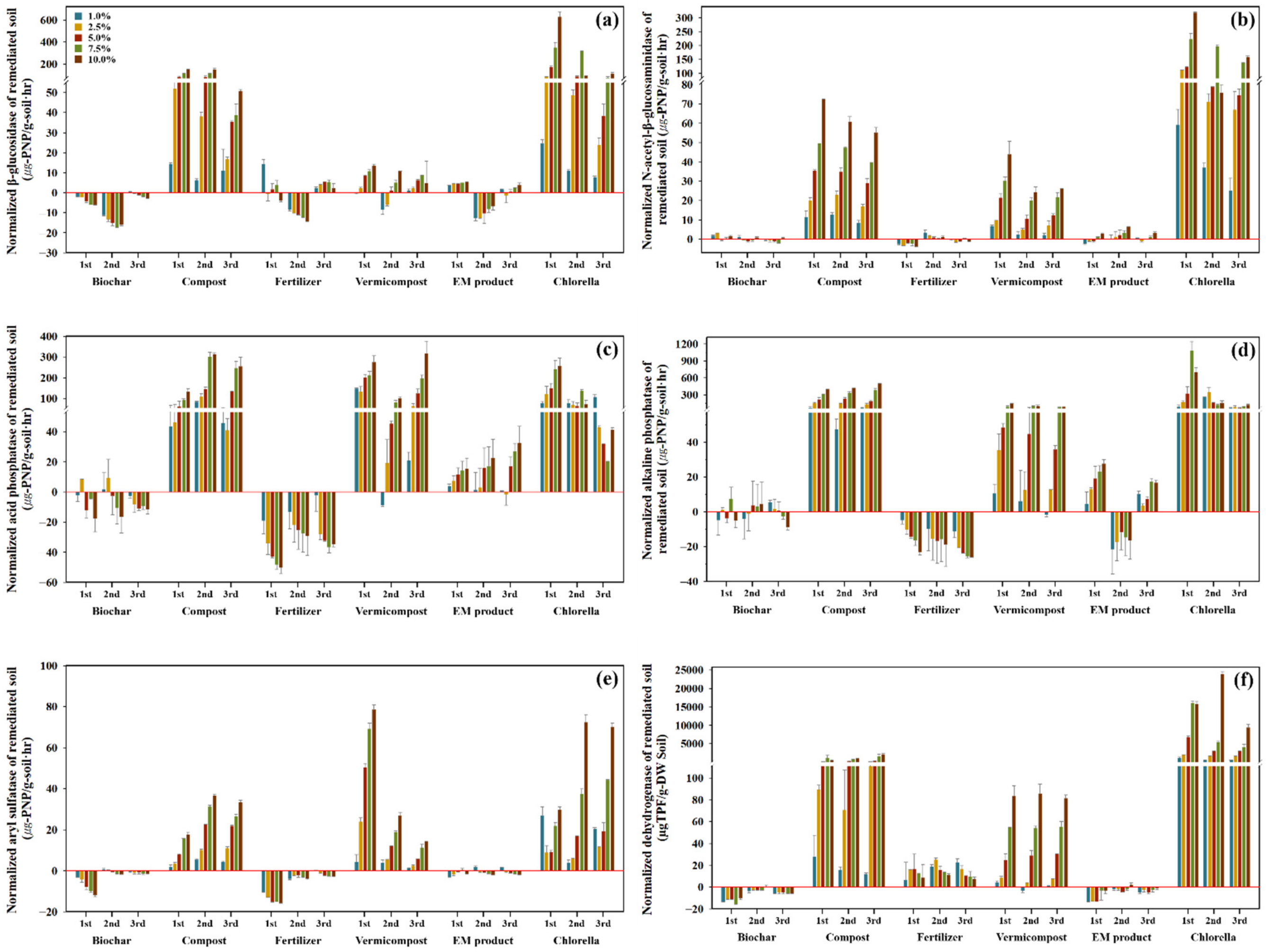

Figure S6. Changes in microbial (soil enzyme activity) factor according to the dosage of each amendment and the duration of treatment. (**a**) β-glucosidase (BG), (**b**) N-acetyl-β-glucosaminidase (NAG), (**c**) acid phosphatase (ACP), (**d**) alkaline phosphatase (ALP), (**e**) arylsulfatase (ARS), and (**f**) dehydrogenase (DHA). The 1st, 2nd, and 3rd periods mean after 2, 6, 10 weeks of treatment. Each result was normalized by subtracting the values of remediated soil (RS) from the values of restored soil.

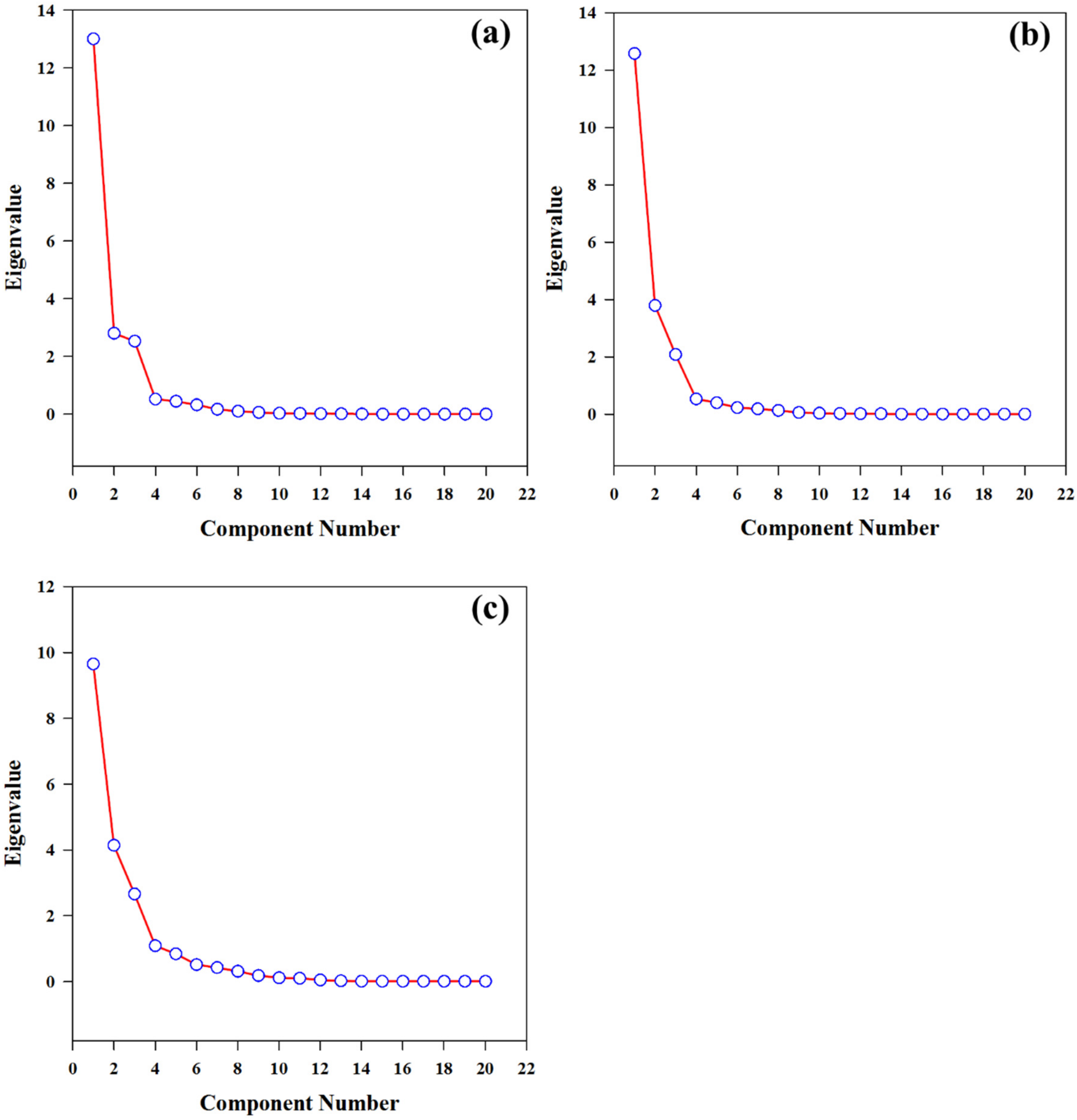

Figure S7. Eigenvalues as a function of component numbers for each amendment obtained from the principal component analyses (PCA). (**a**) compost, (**b**) vermicompost, and (**c**) chlorella.
